# *Streptomyces pallidus* sp. nov. and *Streptomyces qianjiangensis* sp. nov. isolated from the rhizosphere soil of *Cyclosorus acuminatus*

**DOI:** 10.3389/fmicb.2026.1806342

**Published:** 2026-06-08

**Authors:** Ke Mingjun, Huang Wenguang, Tang Ting, Zheng Yaxi, Chen Weijie, Sun Senwei, Cao Yanting, Zhu Le, Mo Ping, Tang Bailu

**Affiliations:** 1 Key Laboratory of Agricultural Products Processing and Food Safety in Hunan Higher Education, Hunan Provincial Engineering Research Center for Fresh Wet Rice Noodles, Science and Technology Innovation Team for Efficient Agricultural Production and Deep Processing at General University in Hunan Province, Changde Key Innovation Team for Wetland Biology and Environmental Ecology, College of Life and Environmental Sciences, Hunan University of Arts and Science, Changde, Hunan, China; 2 College of Furong, Hunan University of Arts and Science, Changde, Hunan, China; 3 College of Synthetic Biology Industry, Hunan University of Arts and Science, Changde, Hunan, China

**Keywords:** ANIm and dDDH, *Cyclosorus acuminatus*, polyphasic taxonomy, *Streptomyces pallidus* sp. nov., *Streptomyces qianjiangensis* sp. nov.

## Abstract

Strains HUAS TT3^T^ and HUAS TT20^T^ were isolated from rhizosphere soil samples of *Cyclosorus acuminatus* collected in Qianjiang, Hubei Province, China. Sequence analyses of the 16S rRNA gene of strains HUAS TT3^T^ and HUAS TT20^T^ indicated that they belonged to the genus *Streptomyces*. Strain HUAS TT3^T^ exhibited the highest sequence similarity of 99.79% to *Streptomyces racemochromogenes* JCM 4407^T^, *Streptomyces polychromogenes* JCM 4505^T^, and *Streptomyces yangpuensis* fd2-11 tb^T^, while strain HUAS TT20^T^ exhibited the highest sequence similarity of 98.76% to *Streptomyces barringtoniae* JA03^T^. Phylogenetic trees constructed using the 16S rRNA gene, five housekeeping genes (*atpD*, *gyrB*, *recA*, *rpoB,* and *trpB*), and whole genome sequences indicated that strain HUAS TT3^T^ is most closely related to *Streptomyces toxytricini* NRRL B-5426^T^ and *Streptomyces globosus* JCM 13859^T^, while strain HUAS TT20^T^ is most closely related to *S. barringtoniae* JA03^T^. However, the average nucleotide identity based on MUMmer (ANIm) and digital DNA–DNA hybridization (dDDH) values were as follows: between HUAS TT3^T^ and *S. toxytricini* JCM 4421^T^, the values were 88.08 and 30.10%; between HUAS TT3^T^ and *S. globosus* JCM 13859^T^, the values were 88.08 and 30.30%; between HUAS TT20^T^ and *S. barringtoniae* JA03^T^, the values were 82.71 and 28.20%. These values were considerably lower than the ANIm and dDDH values for the species boundaries for *Streptomyces*. Moreover, the differential comparisons of phenotypic, cultural, and chemotaxonomic characteristics between HUAS TT3^T^ and *S. toxytricini* CGMCC 4.1734^T^, HUAS TT3^T^ and *S. globosus* JCM 13859^T^, and HUAS TT20^T^ and *S. barringtoniae* LMG 32415^T^ also supported this conclusion. As mentioned above, we conclude that strains HUAS TT3^T^ and HUAS TT20^T^ represent two novel *Streptomyces* species, for which the names *Streptomyces pallidus* sp. nov. HUAS TT3^T^ (MCCC 1K10084^T^ = JCM 38172^T^) and *Streptomyces qianjiangensis* sp. nov. HUAS TT20^T^ (=MCCC 1K10090^T^ = JCM 38175^T^) are proposed.

## 1 Introduction

*Camellia oleifera* is widely distributed around the world and has been cultivated for more than 2,000 years. By 2025, the total planting area of *Camellia oleifera* in China is expected to exceed 6 million hectares, with production capacity for *camellia* oil reaching 2 million tons. Expanding the *camellia* industry across the vast hilly and mountainous regions of southern China is crucial for safeguarding national food and oil security, promoting rural revitalization, and improving public health. However, as the cultivation of *Camellia oleifera* expands, the incidence of oil-tea anthracnose has gradually increased. In numerous provinces and regions, anthracnose causes an annual yield reduction of *camellia* seeds by 10–30%, with severe cases resulting in losses of 40 –50%. In some areas, it can even lead to complete crop failure, resulting in substantial economic losses (Zhuang, 2008).

Currently, the primary strategies for controlling plant diseases can be classified into three categories: breeding disease-resistant varieties, using chemical pesticides, and employing green biocontrol technologies. Among these methods, the rapid development of green biocontrol technologies is the most promising approach. This is because it can provide technical support for the sustainable and efficient development of agricultural production and the safe supply of agricultural products in China. Microbial-based pesticides, which utilize microorganisms, their metabolites, or synthetically modified derivatives of these metabolites, represent a significant international trend in green disease control strategies.

As of 2010, more than 20,000 bioactive compounds derived from microorganisms have been reported worldwide. Among these, microbial bioactive substances produced by actinomycetes accounted for 45%. Notably, approximately 75% (i.e., 7,600 compounds) of these bioactive secondary metabolites from actinomycetes are produced by members of the genus *Streptomyces* (Kaeberlein et al., 2002). Due to their strong antagonistic effects against plant pathogens and other microorganisms, the secondary metabolites from *Streptomyces* have been widely utilized in industry, agriculture, food, and pharmaceuticals (Magarvey et al., 2004; Masand et al., 2015).

The genus *Streptomyces* represents a prominent microbial clade within the phylum Actinomycetota. Since its initial isolation from soil matrices by Waksman and Henrici in the mid-20th century and the subsequent formal establishment of the family Streptomycetaceae in Waksman and Henrici (1943), it has emerged as one of the most speciose genera within the domain *Bacteria*. Advancements in molecular biology techniques have facilitated a re-evaluation of its phylogeny by Stackebrandt et al. (1997). Through the analysis of 16S rRNA sequence similarity and DNA–DNA hybridization data, they proposed the elevation of the family Streptomycetaceae to the suborder Streptomycineae. Subsequent scholarly endeavors have progressively refined the taxonomic framework of this group. Currently, *Streptomyces* serves as the type genus of the family Streptomycetaceae, with *Streptomyces albus* designated as the type strain (Lyons and Pridham, 1962). As of April 2026, the List of Prokaryotic names with Standing in Nomenclature (LPSN) recognizes 810 validly published species within the genus *Streptomyces*. Following continuous and extensive screening efforts, a substantial proportion of compounds derived from terrestrial actinomycetes have been repeatedly rediscovered, leading to a diminishing likelihood of identifying novel bioactive substances and an even lower yield of compounds with potential for drug development. This challenge has become a significant bottleneck in the exploitation of actinomycetes for pharmaceutical applications. Despite this challenge, we maintain that the bioactive compounds discovered in actinomycetes to date represent merely the tip of the iceberg (Hayakawa et al., 2004; Baltz, 2008). To address this limitation, many researchers have shifted their focus to previously underexplored and extreme environments, such as deserts, oceans, intertidal zones, hot springs, saline-alkaline soils, polar regions, caves, and plant-associated habitats, with the aim of applying novel microbial isolation and cultivation techniques. By accessing a broader range of microbial resources, these approaches are expected to enhance the likelihood of discovering new and biologically active compounds (Kaeberlein et al., 2002; Magarvey et al., 2004; Berdy, 2005). In recent years, a growing consensus among scientists suggests that microorganisms from specialized environments likely possess unique metabolic pathways and genetic repertoires (Shomura, 1993). These novel genes have given rise to previously uncharacterized metabolites, positioning such studies as a vital direction for unlocking the potential of microbial resources in drug discovery and biotechnology.

Rhizosphere soil refers to the narrow zone extending approximately 1–4 mm from the root surface, or even less (Dong et al., 2023). Its biological and physical properties are significantly influenced by root exudates and other organic compounds, creating a special microenvironment that differs markedly from that of the bulk soil. It has been reported that microbial population densities in the rhizosphere can be 19–32 times higher than those found in non-rhizosphere soils (Brady et al., 2001). Moreover, actinomycetes isolated from the rhizosphere exhibit a significantly greater ability to produce antimicrobial compounds, indole-3-acetic acid, and other bioactive metabolites compared to those from non-rhizosphere soils (Brady et al., 2002). Therefore, screening for beneficial compounds from rhizosphere-associated actinomycetes represents another promising route for bioprospecting and utilizing natural microbial resources.

Recently, during our screening for actinomycetes that can antagonize *Camellia anthracnose* in the rhizosphere soil of *Cyclosorus acuminatus*, we isolated hundreds of strains from different media using the dilution plate method. Among these strains, two were designated HUAS TT3^T^ and HUAS TT20^T^, both isolated from rhizosphere soil samples of *Cyclosorus acuminatus* collected in Qianjiang, Hubei Province, China (30.363882° N, 112.918033° E). This study focused on the taxonomic identification of HUAS TT3^T^ and HUAS TT20^T^ strains using a polyphasic taxonomy approach.

## 2 Materials and methods

### 2.1 Isolation and maintenance of the organism

Strains HUAS TT3^T^ and HUAS TT20^T^ were isolated from rhizosphere soil samples of *Cyclosorus acuminatus* collected in Qianjiang, Hubei Province, China (30.363882° N, 112.918033° E). The soil samples were heated at 50 °C for 1 h. Strains HUAS TT3^T^ and HUAS TT20^T^ were isolated using the dilution plate method on modified Gause’s synthetic No. 1 medium (Mo et al., 2017), with the addition of a 53.25 mg/L K_2_Cr_2_O_7_ solution to reduce fungal or bacterial contamination. After incubating for 14 days at 28 °C, HUAS TT3^T^ and HUAS TT20^T^ strains were selected and purified on Gause’s synthetic No. 1 medium (Atlas, 1993). For long-term storage, strains HUAS TT3^T^ and HUAS TT20^T^ were preserved in 20% (w/v) glycerol at −20 °C. The type strains *S. toxytricini* CGMCC 4.1734^T^ (CGMCC 4.1804^T^ = DSM 40178^T^ = NBRC 12823^T^) and *S. globosus* CGMCC 4.1969^T^ (DSM 40815^T^ = JCM 13859^T^) were obtained from China General Microbiological Culture Collection Center (CGMCC) in Beijing, China. The type strain *S. barringtoniae* LMG 32415^T^ was acquired from the Laboratorium voor Microbiologie en Microbiele Genetica (LMG) in Rijksuniversiteit, Ledeganckstraat 35, B-9000, Gent, Belgium.

### 2.2 Morphological, cultural, and physiological characteristics

Strains HUAS TT3^T^ and HUAS TT20^T^ were cultured on Gause’s synthetic (Atlas, 1993). After inoculation for 21 days at 28 °C, a Quanta 450 scanning electron microscope (FEI Company, OR, USA) was used to observe spore characteristics. The cultural characteristics of HUAS TT3^T^, *S. toxytricini* CGMCC 4.1734^T^, *S. globosus* CGMCC 4.1969^T^, HUAS TT20^T^, and *S. barringtoniae* LMG 32415^T^ were examined by inoculating these strains on Gause’s synthetic No. 1 medium (Atlas, 1993), Reasoner’s 2A (R2A) (Reasoner and Geldreich, 1985), and International *Streptomyces* Project media 2 through 7 (ISP 2–7) (Shirling and Gottlieb, 1966) for 21 days at 28 °C. By that time, the color standards and color nomenclature (Ridgway, 1912) were used to record the cultural characteristics, including the color of aerial hyphae, substrate mycelia, and soluble pigments. The growth temperature range (4, 10, 15, 20, 25, 28, 30, 35, 40, 45, and 55 °C), sodium chloride (NaCl) tolerance (0, 1, 2, 3, 4, 5, 6, 7, 8, and 9%, w/v) and pH range (3.0, 4.0, 5.0, 6.0, 7.0, 8.0, 9.0, 10.0, 11.0, and 12.0) of strains HUAS TT3^T^, *S. toxytricini* CGMCC 4.1734^T^, *S. globosus* CGMCC 4.1969^T^, HUAS TT20^T,^ and *S. barringtoniae* LMG 32415^T^ were carried out on the ISP 2 agar. The NaCl tolerance and pH range of the tested strains were determined by culturing them for 14 days at 28 °C. The utilization of sole carbon and nitrogen sources, hydrolysis of Tween 20, 40, 60, and 80, hydrolysis of starch, and nitrate reduction were performed as described by Xu et al. (2007). The sole carbon and nitrogen sources were cultured for 6 days at 28 °C and 140 rpm on a rotary shaker. The five strains were cultured under the same conditions for comparison.

### 2.3 Chemotaxonomic characterization

To analyze chemotaxonomically, the strains HUAS TT3^T^, *S. toxytricini* CGMCC 4.1734^T^, *S. globosus* CGMCC 4.1969^T^, HUAS TT20^T,^ and *S. barringtoniae* LMG 32415^T^ were inoculated in tryptic soy broth (TSB) for 6 days at 28 °C, 140 rpm. After growing well, the biomass was collected by centrifugation, and dry cell mass was obtained by vacuum freeze-drying. The fatty acid composition analysis of strains HUAS TT3^T^, *S. toxytricini* CGMCC 4.1734^T^, *S. globosus* CGMCC 4.1969^T^, HUAS TT20^T,^ and *S. barringtoniae* LMG 32415^T^ was carried out by MCCC (Marine Culture Collection of China, Xiamen, China). The isomer of diaminopimelic acid, sugar analyses of whole-cell hydrolysates, and menaquinones of the five strains mentioned above were tested according to the methods described previously (Xu et al., 2007). The polar lipids of strains HUAS TT3^T^ and HUAS TT20^T^ were determined as follows: Dry cell mass (0.3 g) was weighed into a 40 mL centrifuge tube. Next, 2 mL of 1% (w/v) NaCl and 2 mL of sterile distilled water were added, followed by 15 mL of methanol. The tube was tightly capped and heated in a boiling water bath for 1 h with occasional shaking every 10 min to ensure uniform heating. After heating, the mixture was cooled naturally. Chloroform (10 mL) was added, followed by sufficient 2% NaCl to achieve clear phase separation. The mixture was vigorously shaken for 10 min, allowed to stand, and centrifuged. The lower (chloroform) layer was carefully transferred to a round-bottom flask and concentrated to dryness using a rotary evaporator at 40 °C. The dried residue was dissolved in 300 μL of chloroform-methanol (2:1, v/v), and the solution was transferred to a new Eppendorf tube and kept at a low temperature overnight. After overnight storage, the tubes were centrifuged at 8000 rpm for 10 min. If a precipitate formed, the supernatant was collected; if phase separation occurred, the upper layer (foam) was discarded. The remaining material was dried at 50 °C, dissolved in 400 μL of petroleum ether, mixed well, and stored at a low temperature for further use. Polar lipids were separated using two-dimensional thin-layer chromatography (TLC). The sample was spotted onto the plate and developed in the first dimension using chloroform–methanol–water (65:25:4, v/v/v). Then, the plate was developed in the second dimension using chloroform–methanol–acetic acid–water (80:18:12:5, v/v/v/v). Following development, the plates were air-dried and sprayed uniformly with specific detection reagents: ninhydrin reagent (for aminolipids), anisaldehyde reagent (for phosphatidylinositol mannosides), molybdenum blue reagent (for phospholipids), and molybdophosphoric acid reagent (for total lipids). The plates were heated at the following temperatures for color development: 90 °C for ninhydrin, 110 °C for anisaldehyde, 70 °C for molybdenum blue, and 150 °C for molybdophosphoric acid (Xu et al., 2007).

### 2.4 Phylogenetic analysis and genomic DNA–DNA correlation analysis

Genome sequencing of strains HUAS TT3^T^ and HUAS TT20^T^ was performed by Wuhan Benagen Technology Co., Ltd. (Hubei, PR China), according to the methods described by Chen et al. (2022). The full-length 16S rRNA gene sequences of strains HUAS TT3^T^ and HUAS TT20^T^ were obtained from whole-genome sequences, and the full-length 16S rRNA gene sequences of the two strains were separately analyzed using the EzTaxon server (www.ezbiocloud.net/eztaxon) (Yoon et al., 2017). The 16S rRNA gene and five housekeeping gene sequences (*atpD*, *gyrB*, *recA*, *rpoB,* and *trpB*) of the two strains and their relatives were downloaded from the EzTaxon server or National Center for Biotechnology Information (NCBI). The downloaded sequences were used to construct phylogenetic trees using the software package MEGA version 11.0 (Tamura et al., 2021) with 1,000 bootstrap replicates. Neighbor-joining (NJ) (Saitou and Nei, 1987), maximum-likelihood (ML) (Felsenstein, 1981), and Minimum-Evolution (ME) methods (Rzhetsky and Nei, 1992) were used to construct phylogenetic trees using the Kimura 2-parameter model (Kimura, 1980). The phylogenetic tree based on whole-genome sequences was reconstructed using the Type (Strain) Genome Server^1^ (Meier-Kolthoff and Göker, 2019). In order to obtain better results, the whole genome used for reconstructing a phylogenomic tree should meet the criteria of >95% completeness and <5% contamination (Supplementary Table S1) (Parks et al., 2015). The average nucleotide identity based on MUMmer and the digital DNA–DNA hybridization values based on formula d4 between HUAS TT3^T^ and relatives and between HUAS TT20^T^ and relatives were calculated by the online website, the JSpeciesWS service (^2^© 2014–2025 Ribocon GmbH-Version: 5.0.3-Latest system update: 2025-Nov-19-For Research Use Only, Richter et al., 2016), and the genome-to-genome distance calculator (dDDH,^3^ Meier-Kolthof et al., 2013), respectively. NCBI (National Center for Biotechnology Information) and Rapid Annotation using Subsystem Technology (RAST, server version 2.0^4^) were used to analyze and predict the genes of the strains HUAS TT3^T^, *S. toxytricini* JCM 4421^T^, *S. globosus* CGMCC 4.1969^T^, HUAS TT20^T,^ and *S. barringtoniae* LMG 32415^T^ (Aziz et al., 2008). The secondary metabolism biosynthetic gene clusters of the strains were analyzed using antiSMASH version 8.0.4^5^ (Kai et al., 2025).

## 3 Results and discussion

### 3.1 Morphological, cultural, physiological, and biochemical characteristics

**Figure 1:**
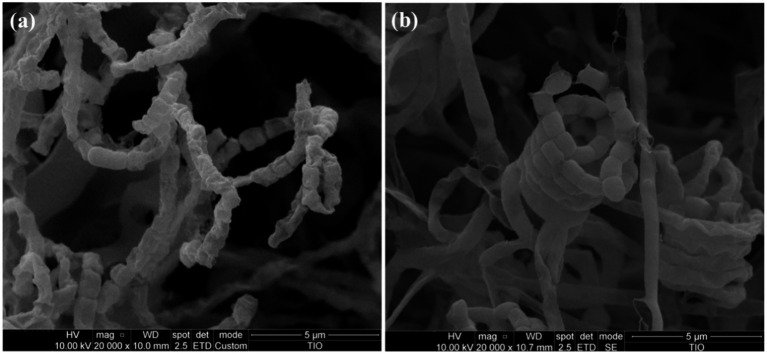
Scanning electron micrographs of strain HUAS TT3^T^ **(a)** and HUAS TT20^T^ **(b)** grown on Gause’s synthetic No. 1 medium at 28 °C after incubation for 21 days.

Strains HUAS TT3^T^ and HUAS TT20^T^ grew well on all tested media. Strain HUAS TT3^T^ produced light-colored or white aerial mycelium and light-colored substrate mycelium on Reasoner’s 2A medium (Supplementary Table S2). Strain HUAS TT20^T^ produced pale gray aerial mycelium and Dresden brown substrate mycelium on Reasoner’s 2A medium (Supplementary Table S3). Strains HUAS TT3^T^ and HUAS TT20^T^ produced flexuous and spiral spore chains, respectively. They produced cylindrical spores with smooth surfaces (Figure 1). Strain HUAS TT3^T^ could grow in 20–35 °C temperature (optimum, 28 °C), tolerance 0–5.0% NaCl (w/t, optimum, 1.0%), and between 5.0–9.0 pH (optimum, 7.0) (Table 1); and strain HUAS TT20^T^ could grow in 20–35 °C temperature (optimum, 28 °C), tolerance 0–3.0% NaCl (w/t, optimum, 1.0%), and between 5.0 and 10.0 pH (optimum, 7.0) (Table 2).

**Table 1:** Differential characteristics of strain HUAS TT3^T^, *S. toxytricini* CGMCC 4.1734^T,^ and *S. globosus* CGMCC 4.1969^T^.

Characteristics	1	2	3
Hydrolysis of starch	−	+	−
Decomposition of Tween 20 and 40	−	+	+
Decomposition of Tween 60 and 80	+	+	−
Nitrate reduction	+	+	−
Growth at/in			
Temperature (°C) (optimum)	20–35 (28)	15–40 (28)	15–35 (28)
Tolerance to NaCl (w/t, optimum)	0–5.0% (1.0%)	0–3.0% (1.0%)	0–2.0% (1.0%)
pH (optimum)	5.0–9.0 (7.0)	6.0–10.0 (7.0)	6.0–10.0 (7.0)
Carbon utilization			
d-Mannose	+	−	−
d-Ribose	−	+	+
Fructose	+	−	−
Glucose	−	+	+
Lactose	−	+	−
l-Arabinose	+	+	−
l-Rhamnose	−	+	−
Mannitol	−	+	+
Inositol	−	−	+
Nitrogen utilization			
l-Alanine	−	+	−
l-Arginine	+	+	−
l-Cysteine	−	+	−
l-Glutamine	−	−	+
l-Glycine	+	+	−
l-Histidine	−	+	+
l-Hydroxyproline	−	+	+
l-Serine	−	−	+
l-Ornithine	+	−	+
l-Proline	−	+	+
l-Valine	+	+	−
Menaquinones	MK-9(H_4_) (30.2%) MK-9(H_6_) (43.2%) MK-9(H_8_) (25.6%)	MK-9(H_4_) (20.5%) MK-9(H_6_) (56.8%) MK-9(H_8_) (21.3%)	MK-9(H_4_) (22.3%) MK-9(H_6_) (50.6%) MK-9(H_8_) (16.4%)
Whole-cell sugars	Glucose, mannose, ribose	Glucose, ribose	Glucose, ribose
Cell wall diamino acid	*ll*-DAP	*ll*-DAP	*ll*-DAP

Strains: 1, HUAS TT3^T^; 2, *S. toxytricini* CGMCC 4.1734^T^; 3, *S. globosus* CGMCC 4.1969^T^. +, positive; −, negative. All data were obtained from this study. Strains 1, 2 and 3 utilized cellobiose as the sole carbon source; l-arginine, l-leucine, and l-phenylalanine as sole nitrogen sources; d-galactose, sucrose, or xylose as sole carbon sources; and l-asparagine, l-tyrosine, and methionine as sole nitrogen sources.

**Table 2:** Differential characteristics of strain HUAS TT20^T^ and *S. barringtoniae* LMG 32415^T^.

Characteristics	1	2
Hydrolysis of starch	−	−
Nitrate reduction	+	+
Decomposition of Tween 20 and 40	−	+
Decomposition of Tween 60 and 80	−	+
Growth at/in		
Temperature (°C) (optimum)	20–35 °C (28 °C)	25–35 °C (28 °C)
Tolerance to NaCl (optimum)	0–3.0% (1.0%)	0–6.0% (1.0%)
pH (optimum)	5.0–10.0 (7.0)	5.0–9.0 (7.0)
Carbon utilization		
d-Mannose	−	+
Fructose	+	−
Glucose	+	−
l-Arabinose	+	−
l-Rhamnose	−	+
Sucrose	+	−
Nitrogen utilization		
l-Alanine	+	−
l-Glycine	−	+
l-Histidine	+	−
l-Ornithine	−	+
l-Phenylalanine	−	+
l-Tyrosine	+	−
Methionine	−	+
Menaquinones	MK-9(H_2_) (44.2%) MK-9(H_6_) (18.5%) MK-9(H_8_) (35.7%)	MK-9(H_4_) (18.9%) MK-9(H_6_) (45.7%) MK-9(H_8_) (32.8%)
Whole-cell sugars	Mannose, ribose	Glucose, ribose
Cell wall diamino acid	*ll*-DAP	*ll*-DAP

Strains: 1, HUAS TT20^T^; 2, *S. barringtoniae* LMG 32415^T^. +, positive; −, negative. All data were from this study. Two strains were positive for nitrate reduction but negative for starch hydrolysis. Strains 1 and 2 utilized cellobiose and d-galactose as sole carbon sources and l-arginine, l-asparagine, l-cysteine, l-leucine, l-proline, and l-valine as sole nitrogen sources. However, d-ribose, inositol, lactose, mannitol, and xylose are used as sole carbon sources, and l-glutamine, l-hydroxyproline, and l-serine are not utilized as sole nitrogen sources.

### 3.2 Chemotaxonomic characteristics

The polar lipid composition of strain HUAS TT3^T^ was diphosphatidylglycerol (DPG), phosphatidylethanolamine (PE), phosphatidylglycerol (PG), phosphatidylinositol (PI), and phosphatidylinositol mannosides (PIM); and strain HUAS TT20^T^ consisted of diphosphatidylglycerol (DPG), phosphatidyl choline (PC), phosphatidylethanolamine (PE), phosphatidylglycerol (PG), unidentified phospholipid (PL), phosphatidylinositol (PI), and phosphatidylinositol mannosides (PIM) (Supplementary Figure S1). The major fatty acids of strain HUAS TT3^T^ (>5.0%) were C_16:1_ ω5c (15.6%), C_17:1_ ω8c (10.2%), *iso*-C_16:0_ (9.3%), *anteiso*-C_14:0_ (8.6%), and C_15:0_ (6.7%) (Supplementary Table S4); and the composition of strain HUAS TT20^T^ (>5.0%) were *iso*-C_16:0_ (22.9%), *iso*-C_14:0_ (17.1%), C_17:0_ (10.0%), *anteiso*-C_15:0_ (8.7%), C_16:0_ (7.1%), *iso-*C_15:0_ (5.6%) and Summed Feature 3 (C_16:1_ ω7c/C_16:1_ ω6c, 5.4%) (Supplementary Table S5). The menaquinones of strain HUAS TT3^T^ were MK-9(H_4_) (30.2%), MK-9(H_6_) (43.2%), and MK-9(H_8_) (25.6%), and those of strain HUAS TT20^T^ were MK-9(H_2_) (44.2%), MK-9(H_8_) (35.7%), and MK-9(H_6_) (18.5%). The cell walls of HUAS TT3^T^ and HUAS TT20^T^ contained ll-diaminopimelic acid (ll-DAP). The whole-cell hydrolysates of strain HUAS TT3^T^ contained glucose, mannose, and ribose, and those of strain HUAS TT20^T^ contained mannose and ribose (Tables 1, 2).

### 3.3 Genomic analysis

The DNA G + C content of the genome sequence of strain HUAS TT3^T^, containing 7,793,067 bp, was 73.0%, and that of strain HUAS TT20^T^, containing 9,258,818 bp, was 70.5%. Strain HUAS TT3^T^ contained a total of 7,648 genes (7,286 coding genes, 100 RNA genes, and 262 pseudogenes), 7,548 Coding DNA Sequences (CDSs) (7,286 CDSs with protein and 262 CDSs without protein); and strain HUAS TT20^T^ contained a total of 8,788 genes (8,145 coding genes, 95 RNA genes, and 548 pseudogenes), 8,693 CDSs (8,145 CDSs with protein and 548 CDSs without protein) (Supplementary Tables S6, S7). Strain HUAS TT3^T,^ as annotated by the RAST web-service consisted of ‘amino acids and derivatives’ (398 CDSs), ‘carbohydrates’ (326 CDSs), ‘protein metabolism’ (247 CDSs), ‘cofactors, vitamins, prosthetic groups, and pigments’ (188 CDSs), ‘fatty acids, lipids, and isoprenoids’ (167 CDSs), ‘respiration’ (115 CDSs), ‘DNA metabolism’ (90 CDSs), ‘nucleosides and nucleotides’ (114 CDSs), and so on; and strain HUAS TT20^T^ consisted of ‘amino acids and derivatives’ (452 CDSs), ‘carbohydrates’ (407 CDSs), ‘protein metabolism’ (247 CDSs), ‘cofactors, vitamins, prosthetic groups, and pigments’ (213 CDSs), ‘fatty acids, lipids, and isoprenoids’ (224 CDSs), ‘respiration’ (146 CDSs), ‘DNA metabolism’ (113 CDSs), ‘nucleosides and nucleotides’ (107 CDSs), and so on (Supplementary Tables S8, S9). The results of the AntiSMASH analysis indicated that the genome of strain HUAS TT3^T^ consisted of four main biosynthetic gene clusters: terpene, T1PKS (Type I PKS, Polyketide synthase), NRPS (non-ribosomal peptide synthetase), and NRPS-like (NRPS-like fragment), whereas strain HUAS TT20^T^ consisted of terpene, T1PKS (Type I PKS, Polyketide synthase), NI-siderophore [NRPS-independent, IucA/IucC-like siderophores (siderophores before 7.0)], and NRPS (non-ribosomal peptide synthetase) (Supplementary Tables S10, S11).

### 3.4 Phylogeny and DNA–DNA correlation analysis

**Figure 2:**
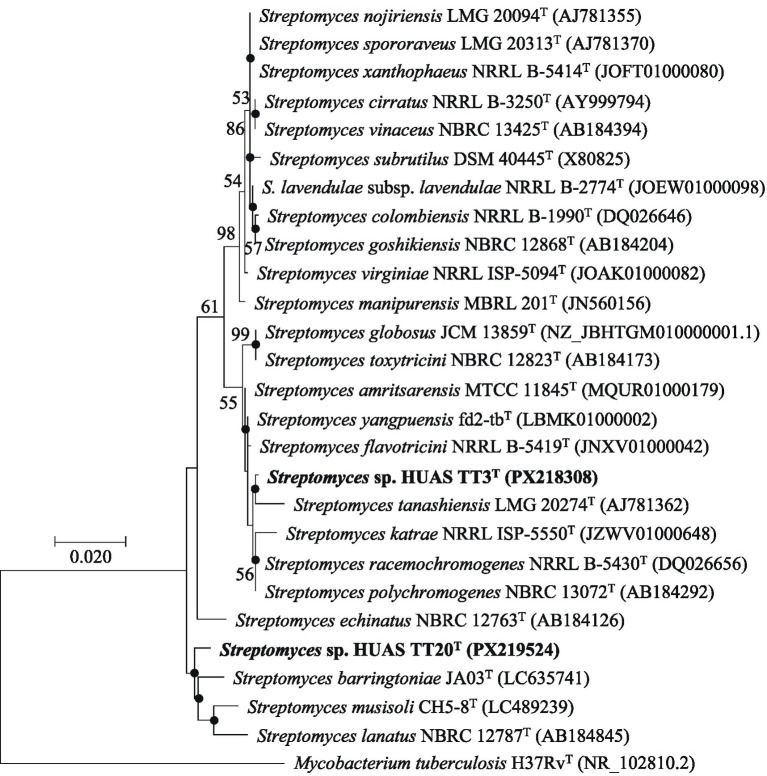
Maximum-likelihood phylogenetic tree based on 16S rRNA gene sequences showing the relationship between strains HUAS TT3^T^ and HUAS TT20^T^ and selected species of the genus *Streptomyces*. *Mycobacterium tuberculosis* H37Rv^T^ was used as an outgroup. Bootstrap percentages over 50% derived from 1,000 replications are shown at the nodes. Dots indicate that branches were also recovered in the neighbor-joining and maximum-parsimony trees. Scale bar: 0.020 nucleotides per site.

**Figure 3:**
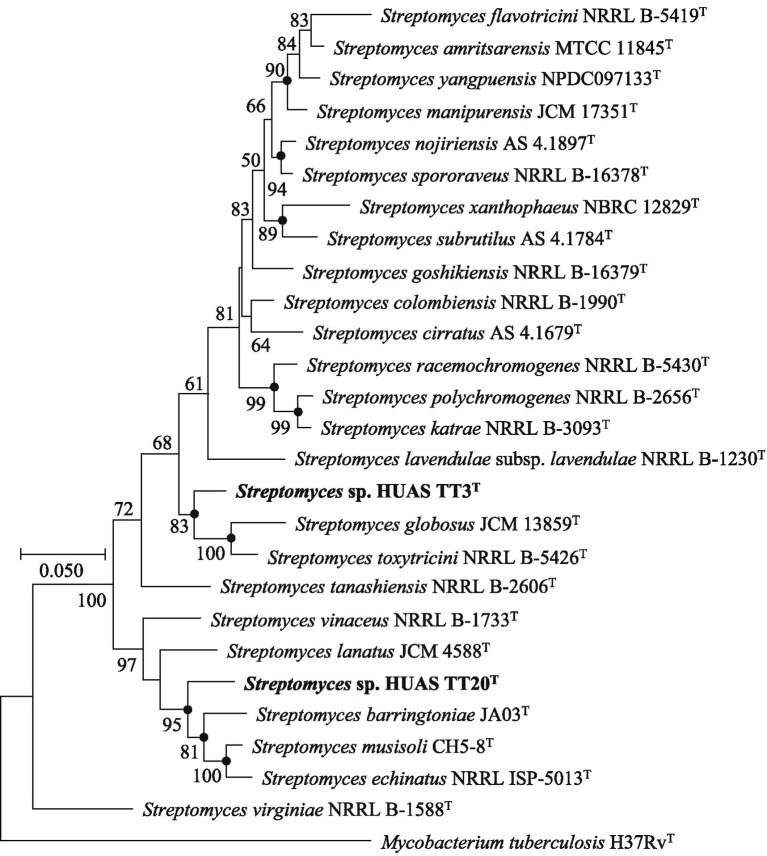
Maximum-likelihood phylogenetic tree based on five housekeeping gene sequences showing the relationship between strain HUAS TT3^T^, HUAS TT20^T,^ and selected *Streptomyces* species. *Mycobacterium tuberculosis* H37Rv^T^ was used as the outgroup. Bootstrap percentages of over 50% derived from 1,000 replications are shown at the nodes. Dots indicate that branches were also recovered in the neighbor-joining and maximum-parsimony trees. Scale bar, 0.05 substitutions per site.

Sequence analyses of the 16S rRNA gene of strains HUAS TT3^T^ (1528 bp) and HUAS TT20^T^ indicated that they belonged to the genus *Streptomyces*. Then strain HUAS TT3^T^ exhibited similarities to *Streptomyces racemochromogenes* JCM 4407^T^ (99.79%), *Streptomyces polychromogenes* JCM 4505^T^ (99.79%), *Streptomyces yangpuensis* fd2-tb^T^ (99.79%), *Streptomyces flavotricini* NRRL B-5419^T^ (99.72%), *Streptomyces amritsarensis* Microbial Type Culture Collection and Gene Bank (MTCC) 11845^T^ (99.65%), *Streptomyces globosus* JCM 13859^T^ (99.31%), *Streptomyces toxytricini* JCM 4421^T^ (99.31%), *Streptomyces katrae* NRRL ISP-5550^T^ (99.24%), *Streptomyces virginiae* DSM 40803^T^ (99.03%), *Streptomyces tanashiensis* JCM 4086^T^ (98.96%), *Streptomyces manipurensis* JCM 17351^T^ (98.89%), *Streptomyces lavendulae* subsp. *lavendulae* NRRL B-2774^T^ (98.76%), *Streptomyces xanthophaeus* NBRC 12829^T^ (98.76%), *Streptomyces nojiriensis* JCM 3382^T^ (98.76%), *Streptomyces spororaveus* NBRC 15456^T^ (98.76%), *Streptomyces colombiensis* NRRL B-1990^T^ (98.69%), *Streptomyces cirratus* JCM 4738^T^ (98.69%), *Streptomyces vinaceus* ATCC 27476^T^ (98.68%), *Streptomyces goshikiensis* JCM 4640^T^ (98.67%), and lower sequence similarities to other type species of genus *Streptomyces*; while the 16S rRNA gene of HUAS TT20^T^ showed similarities to *Streptomyces barringtoniae* JA03^T^ (98.76%), and *Streptomyces musisoli* CH5-8^T^ (98.52%). The ML, NJ, and ME phylogenetic trees based on the 16S rRNA gene sequences indicated that strain HUAS TT3^T^ was most closely related to *S. toxytricini* NRRL B-5426^T^ and *S. globosus* JCM 13859^T^, and strain HUAS TT20^T^ was most closely related to *S. barringtoniae* JA03^T^ (Figure 2, Supplementary Figures S2, S3). The ML, NJ, and ME phylogenetic trees based on the five housekeeping genes (*atpD*, *gyrB*, *recA*, *rpoB,* and *trpB*) also supported this result (Figure 3, Supplementary Figures S4, S5). Phylogenetic analyses based on whole genome sequences also further support this conclusion (Supplementary Figure S6). However, the average nucleotide identity based on MUMmer (ANIm) and the digital DNA–DNA hybridization (dDDH) values between HUAS TT3^T^ and *S. toxytricini* JCM 4421^T^, HUAS TT3^T,^ and *S. globosus* JCM 13859^T^, and between HUAS TT20^T^ and *S. barringtoniae* JA03^T^ were 88.08 and 30.10%, 88.08, and 30.30%, 82.71 and 28.20%, respectively, far less than the 96.7% ANIm and 70% dDDH as species boundaries for *Streptomyces* (Hu et al., 2022; Wayne et al., 1987). As Stackebrandt and Ebers (2006) pointed out, if the 16S rRNA gene sequence similarity between two strains is ≥98.7%, ANI and dDDH or DDH values should be calculated to evaluate whether it represents a new species. In this work, considering that the HUAS TT3^T^ shares ≥98.7% 16S rRNA gene sequence similarities to strains, *S. racemochromogenes* JCM 4407^T^, *S. polychromogenes* JCM 4505^T^, *S. yangpuensis* fd2-tb^T^, *S. flavotricini* NRRL B-5419^T^, *S. amritsarensis* MTCC 11845^T^, *S. katrae* NRRL ISP-5550^T^, *Streptomyces virginiae* DSM 40803^T^, *S. tanashiensis* JCM 4086^T^, *S. manipurensis* JCM 17351^T^, *S. lavendulae* subsp. *Lavandulae* NRRL B-2774^T^, *S. xanthophaeus* NBRC 12829^T^, *S. nojiriensis* JCM 3382^T^, *S. spororaveus* NBRC 15456^T^, *S. colombiensis* NRRL B-1990^T^, *S. cirratus* JCM 4738^T^, *S. vinaceus* ATCC 27476^T^, and *S. goshikiensis* JCM 4640^T^. Therefore, ANIm and dDDH between strain HUAS TT3^T^ and the above-mentioned strains should be calculated. The ANIm and dDDH between them were 85.80 –87.95%, 23.30 –30.10%, and 79.65 –85.15% (Table 3), respectively, far less than the 96.7% ANIm and 70% dDDH as species boundaries for *Streptomyces* (Hu et al., 2022; Wayne et al., 1987). Due to the lack of a whole genome for strain *S. colombiensis* NRRL B-1990^T^, the ANI and dDDH values between HUAS TT3^T^ and strain *S. colombiensis* NRRL B-1990^T^ could not be calculated. However, according to Hu et al. (2022), multilocus sequence analysis (MLSA) based on housekeeping genes (*atpD*, *gyrB*, *recA*, *rpoB,* and *trpB*) has been proven to be an alternative and effective method for identifying *Streptomyces* species. However, the pairwise multilocus sequence analysis (MLSA) distance between strain HUAS TT3^T^ and *S. colombiensis* NRRL B-1990^T^ was 0.073 (Supplementary Tables S12, S13), much higher than the maximum range of 0.014 recommended for delineating *Streptomyces* species (Hu et al., 2022). These results further affirmed that strains HUAS TT3^T^ and HUAS TT20^T^ represent two novel *Streptomyces* species. Moreover, the differential comparisons of the phenotypic, cultural, and chemotaxonomic characteristics between HUAS TT3^T^ and *S. toxytricini* CGMCC 4.1734^T^, HUAS TT3^T^ and *S. globosus* CGMCC 4.1969^T^, and HUAS TT20^T^ and *S. barringtoniae* LMG 32415^T^ again supported this conclusion. For example, strain HUAS TT3^T^ was utilized as d-mannose and fructose for sole carbon sources but not as mannitol for sole carbon sources. However, *S. toxytricini* CGMCC 4.1734^T^ and *S. globosus* CGMCC 4.1969^T^ are in contrast. Strain HUAS TT20^T^ was utilized as l-alanine, l-histidine, and l-tyrosine for sole nitrogen sources, but *S. barringtoniae* LMG 32415^T^ is not. Detailed descriptions are shown in Tables 1, 2 and Supplementary Tables S2–S11.

**Table 3:** Comparative genotypic analysis between strain HUAS TT3^T^ and the closest related type strains, *S. racemochromogenes* JCM 4407^T^, *S. polychromogenes* JCM 4505^T^, *S. yangpuensis* fd2-tb^T^, *S. flavotricini* NGL1^T^, *S. amritsarensis* MTCC 11845^T^, *S. globosus* JCM 13859^T^, *S. toxytricini* JCM 4421^T^, *S. katrae* NRRL ISP-5550^T^, *S. virginiae* DSM 40803^T^, *S. tanashiensis* JCM 4086^T^, *S. manipurensis* JCM 17351^T^, *S. lavendulae* subsp*. lavendulae* NRRL B-2774^T^, *S. xanthophaeus* NBRC 12829^T^, *S. nojiriensis* JCM 3382^T^, *S. spororaveus* NBRC 15456^T^, *S. cirratus* JCM 4738^T^, *S. vinaceus* ATCC 27476^T^, and *S. goshikiensis* JCM 4640^T^.

Closest related species	Strain HUAS TT3^T^		
	16S rRNA gene sequence similarity	ANIm	dDDH
*S. racemochromogenes* JCM 4407^T^	99.79%	87.48%	28.30%
*S. polychromogenes* JCM 4505^T^	99.79%	87.35%	27.60%
*S. yangpuensis* fd2-tb^T^	99.72%	87.35%	28.10%
*S. flavotricini* NRRL B-5419^T^	99.65%	87.54%	29.20%
*S. amritsarensis* MTCC 11845^T^	99.65%	87.32%	28.30%
*S. globosus* JCM 13859^T^	99.31%	88.08%	30.30%
*S. toxytricini* JCM 4421^T^	99.31%	88.08%	30.10%
*S. katrae* NRRL ISP-5550^T^	99.24%	87.40%	28.20%
*S. virginiae* DSM 40803^T^	99.03%	87.35%	28.10%
*S. tanashiensis* JCM 4086^T^	98.96%	85.80%	23.30%
*S. manipurensis* JCM 17351^T^	98.89%	87.25%	27.70%
*S. lavendulae* subsp*. lavendulae* NRRL B-2774^T^	98.76%	87.23%	27.80%
*S. xanthophaeus* NBRC 12829^T^	98.76%	87.25%	28.00%
*S. nojiriensis* JCM 3382^T^	98.76%	87.43%	28.40%
*S. spororaveus* NBRC 15456^T^	98.76%	87.31%	27.90%
*S. colombiensis* NRRL B-1990^T^	98.69%	ND	ND
*S. cirratus* JCM 4738^T^	98.69%	87.02%	27.40%
*S. vinaceus* ATCC 27476^T^	98.68%	87.95%	30.10%
*S. goshikiensis* JCM 4640^T^	98.67%	87.33%	28.10%

ND, not determined.

In conclusion, based on phenotypic and chemotaxonomic characteristics, as well as phylogeny, ANI, dDDH, and MLSA data, strains HUAS TT3^T^ and HUAS TT20^T^ represent two novel *Streptomyces* species, for which the names *Streptomyces pallidus* sp. nov. and *Streptomyces qianjiangensis* sp. nov. are proposed.

## 4 Description

### 4.1 Description of *Streptomyces pallidus* sp. nov.

*Streptomyces pallidus* (pal’li.dus. L. masc. Adj. pallidus, pale).

It grows well on all tested media and produces diffusible pigments. It produces light-colored or white aerial mycelium and light-colored substrate mycelium on Reasoner’s 2A medium. It produces flexuous chains of cylindrical spores with smooth surfaces (0.642–0.771 × 0.702–1.560 μm). It is positive for nitrate reduction and decomposition of Tween 60 and 80 but negative for hydrolysis of starch and decomposition of Tween 20 and 40. It grows at 20–35 °C (optimum, 28 °C), with tolerance to 0–5.0% NaCl (w/t, optimum, 1.0%), and between 5.0 and 9.0 pH (optimum, 7.0). It utilizes cellobiose, d-mannose, fructose, and l-arabinose as the sole carbon sources, but not d-galactose, d-ribose, glucose, inositol, lactose, l-rhamnose, mannitol, sucrose, or xylose. It utilizes l-arginine, l-glycine, l-leucine, l-ornithine, l-phenylalanine, and l-valine as the sole nitrogen sources, but not l-alanine, l-asparagine, l-cysteine, l-glutamine, l-histidine, l-hydroxyproline, l-proline, l-serine, l-tyrosine, or methionine.

The polar lipids are diphosphatidylglycerol (DPG), phosphatidylethanolamine (PE), phosphatidylglycerol (PG), phosphatidylinositol (PI), and phosphatidylinositol mannosides (PIM). The major fatty acids are C_16:1_ ω5c (15.6%), C_17:1_ ω8c (10.2%), *iso*-C_16:0_, *anteiso*-C_14:0_, and C_15:0_. The menaquinones are MK-9(H_4_), MK-9(H_6_), and MK-9(H_8_). The cell wall contains ll-diaminopimelic acid (ll-DAP), and the whole-cell hydrolysates are glucose, mannose, and ribose.

The type strain, HUAS TT3^T^ = (MCCC 1K10084^T^ = JCM 38172^T^), was isolated from rhizosphere soil samples of *Cyclosorus acuminatus* collected from Qianjiang, Hubei Province, PR China. The DNA G + C content of the genome sequence, containing 7,793,067 bp, was 73.0%. A total of 7,648 genes (7,286 coding genes, 100 RNA genes, and 262 pseudogenes) and 7,548 CDSs (7,286 CDSs with protein and 262 CDSs without protein) were predicted. The NCBI accession numbers for the 16S rRNA gene and genome sequence of HUAS TT3^T^ are PX218308 and CP196181.1.

### 4.2 Description of *Streptomyces qianjiangensis* sp. nov.

*Streptomyces qianjiangensis* (qian.ji.ang.en’sis. N. L. masc. Adj. *qianjiangensis*, about Qianjiang, a city in Hubei Province, PR China).

It produces spiral spore chains and cylindrical spores with smooth surfaces (0.639–0.745 × 0.861–1.198 μm). It grows well on all tested media and produces diffusible pigments on Reasoner’s 2A, ISP 6, and 7. It produces pale gray aerial mycelia and Dresden brown substrate mycelia on Reasoner’s 2A medium. It produces spiral spore chains and cylindrical spores with smooth surfaces. Positive for nitrate reduction but negative for hydrolysis of starch and decomposition of Tween 20, 40, 60, and 80. It grows at 20–35 °C temperature (optimum, 28 °C), tolerance to 0–3.0% NaCl (w/t, optimum, 1.0%), and between 5.0 and 10.0 pH (optimum, 7.0). It utilizes cellobiose, d-galactose, fructose, glucose, l-arabinose, and sucrose as the sole carbon sources, but not d-mannose, d-ribose, inositol, lactose, l-rhamnose, mannitol, or xylose. It utilizes l-alanine, l-arginine, l-asparagine, l-cysteine, l-histidine, l-leucine, l-proline, l-tyrosine, and l-valine as the sole nitrogen sources, but not l-glutamine, l-glycine, l-hydroxyproline, l-ornithine, l-phenylalanine, l-serine, and methionine. Polar lipids include diphosphatidylglycerol (DPG), phosphatidylcholine (PC), phosphatidylethanolamine (PE), phosphatidylglycerol (PG), unidentified phospholipids (PL), phosphatidylinositol (PI), and phosphatidylinositol mannosides (PIM). The major fatty acids (>5.0%) are *iso*-C_16:0_, *iso*-C_14:0_, C_17:0_, *anteiso*-C_15:0_, C_16:0_, *iso-*C_15:0,_ and Summed Feature 3 (C_16:1_ ω7c/C_16:1_ ω6c). The menaquinones are MK-9(H_2_), MK-9(H_8_), and MK-9(H_6_). The cell wall contains ll-diaminopimelic acid (ll-DAP), and the whole-cell hydrolysates are mannose and ribose.

The type strain HUAS TT20^T^ = MCCC 1K10090^T^ = JCM 38175^T^ was isolated from rhizosphere soil samples of *Cyclosorus acuminatus* collected from Qianjiang, Hubei Province, PR China. The DNA G + C content of the genome sequence of strain HUAS TT20^T^, containing 9,258,818 bp, was 70.5%. A total of 8,788 genes (8,145 coding genes, 95 RNA genes, and 548 pseudogenes) and 8,693 CDSs (8,145 CDSs with protein and 548 CDSs without protein) were predicted. The NCBI accession numbers for the 16S rRNA gene sequence and genome sequence of HUAS TT20^T^ are PX219524 and CP106798.1, respectively.

## Data Availability

The datasets presented in this study can be found in online repositories. The names of the repository/repositories and accession number(s) can be found in the article/Supplementary material.

## Footnotes

1
https://tygs.dsmz.de/

2
https://jspecies.ribohost.com/jspeciesws/#analyse

3
http://ggdc.dsmz.de/distcalc2.php

4
https://rast.nmpdr.org

5
https://antismash.secondarymetabolites.org
