# Supplementary material for: Streptomyces pallidus sp. nov. and Streptomyces qianjiangensis sp. nov. isolated from the rhizosphere soil of Cyclosorus acuminatus
Source: Front Microbiol. 2026 Jun 8;17:1806342. doi: 10.3389/fmicb.2026.1806342 (PMC13283956; doi:10.3389/fmicb.2026.1806342)
Supplement: Supplementary file 1 [file Supplementary_file_1.pdf]

Streptomyces pallidus* sp. nov., and *Streptomyces qianjiangensis* sp. nov., isolated from the rhizosphere soil of *Cyclosorus acuminatus

Ke Mingjun [1†], Huang Wenguang [1, 2†], Tang Ting [1], Zheng Yaxi [1], Chen Weijie [1, 2], Sun Senwei [1], Cao Yanting [1], Zhu Le [1], Mo Ping [1, 2, 3*], and Tang Bailu [1*]

Author affiliations:

[1]Key Laboratory of Agricultural Products Processing and Food Safety in Hunan Higher Education, Hunan Provincial Engineering Research Center for Fresh Wet Rice Noodles, Science and Technology Innovation Team for Efficient Agricultural Production and Deep Processing at General University in Hunan Province, Changde Key Innovation Team for wetland biology and Environmental Ecology, College of Life and Environmental Sciences, Hunan University of Arts and Science, Changde 415000, Hunan Province, People's Republic of China.

[2]College of Furong, Hunan University of Arts and Science, Changde 415000, Hunan Province, People's Republic of China.

[3]College of Synthetic Biology Industry, Hunan University of Arts and Science, Changde 415000, Hunan Province, People's Republic of China.

[*]Correspondence:

Mo Ping, moping2015@126.com; Tang Bailu, tangbl@huas.edu.cn

†These authors contributed equally to this work.

Fig. S1. Polar lipids composition of strain HUAS TT3^T and HUAS TT20^T.

The plate dotted with sample was subjected to two-dimensional development, with the first solvent of chloroform-methanol-water (65:25:4, v/v/v) followed by the second solvent of chloroform-methanol-acetic acid-water (80:18:12:5, v/v/v/v).

Note: 1, HUAS TT3^T; HUAS TT20. Molybdophosphoric acid, molybdenum blue reagent, anisaldehyde and ninhydrin were used to detect total lipids, phospholipids, phosphatidylinositol mannosides and aminolipids respectively. A, Molybdophosphoric acid (for total lipids); B, Molybdenum blue reagent (for phospholipids); C, Anisaldehyde (for phosphatidylinositol mannosides); D, Ninhydrin (for aminolipids). DPG, diphosphatidylglycerol; PC, phosphatidyl choline; PE, phosphatidyl ethanolamine; PG, phosphatidylglycerol; PL, unidentified phospholipid; PI, phosphotidylinositol; PIM, phosphatidylinositol mannosides.

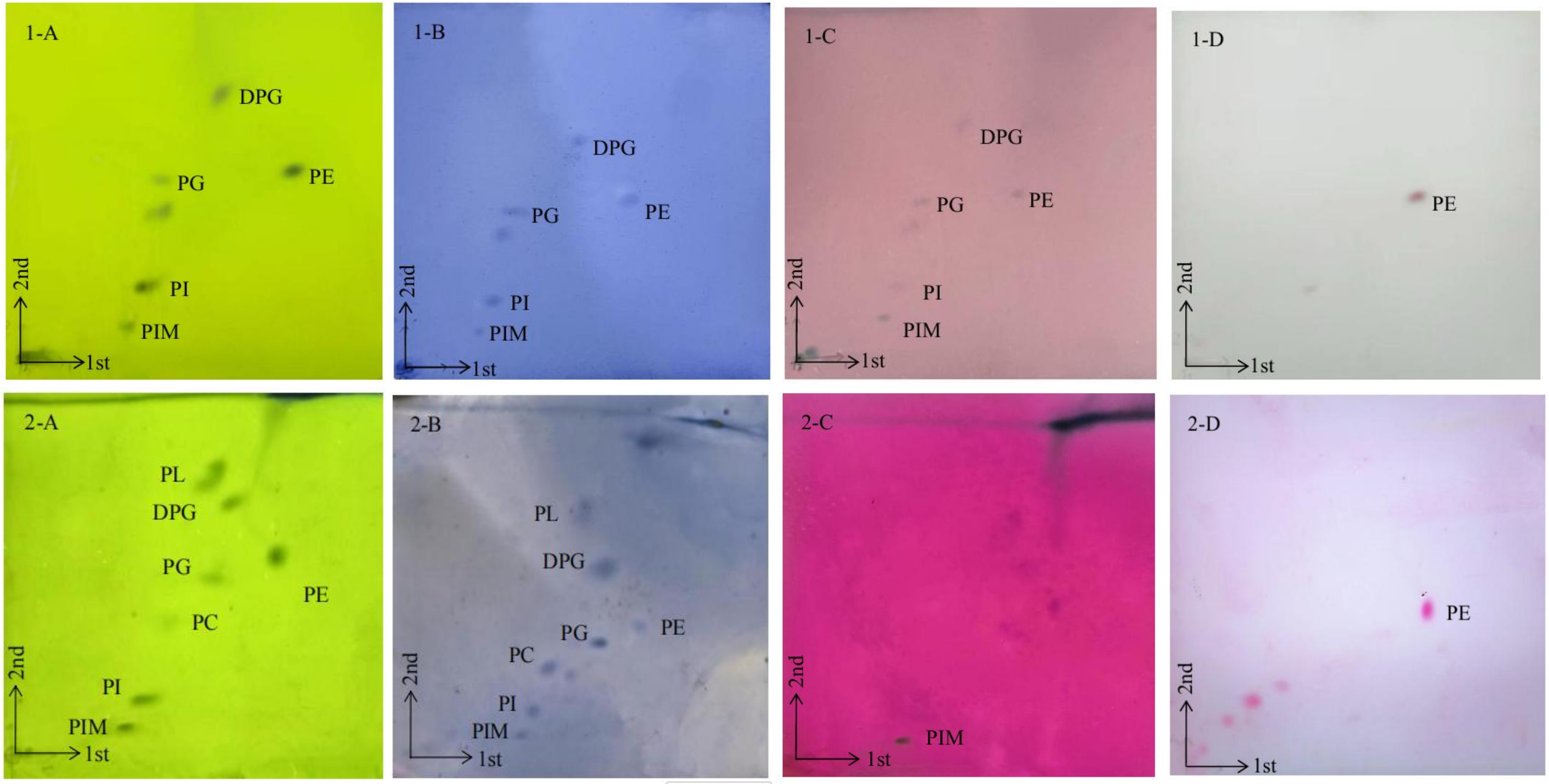
1-A
DPG
PG
PE
PI
PIM
2nd
1st
1-B
DPG
PG
PE
PI
PIM
2nd
1st
1-C
DPG
PG
PE
PI
PIM
2nd
1st
1-D
PE
2nd
1st
2-A
PL
DPG
PG
PE
PC
PI
PIM
2nd
1st
2-B
PL
DPG
PG
PE
PC
PI
PIM
2nd
1st
2-C
PIM
2nd
1st
2-D
PE
2nd
1st

Fig. S2. Neighbour-joining phylogenetic tree based on 16S rRNA gene sequences showing the relationship between strain HUAS TT3^T, HUAS TT20^T and selected species of the genus *Streptomyces*. Mycobact*erium tuberculosis* H37Rv^T was used as an outgroup. Bootstrap percentages over 50% derived from 1000 replications are shown at the nodes. Scale bar, 0.0050 substitutions per site.

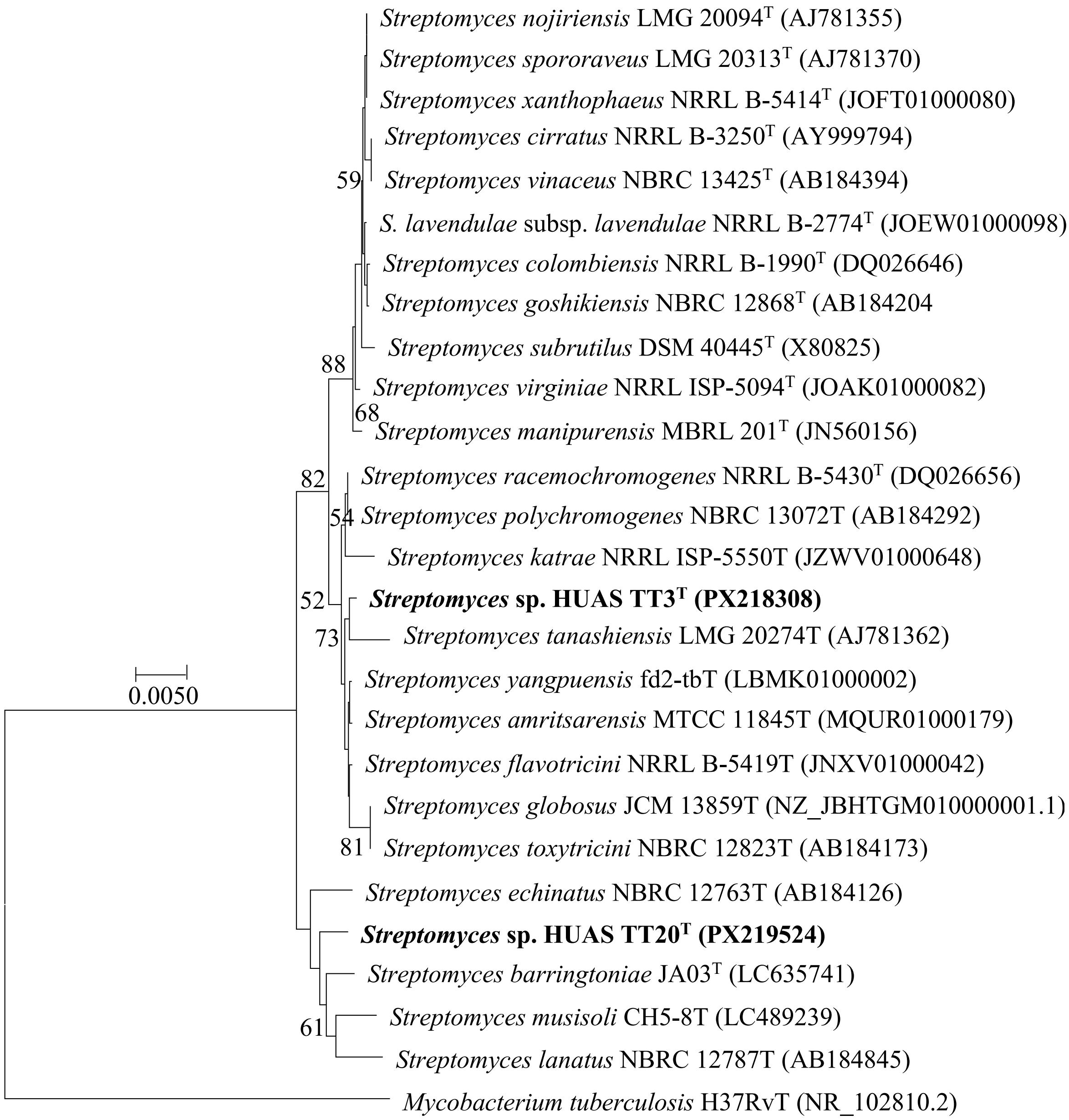

Fig. S3. Minimum evolution phylogenetic tree based on 16S rRNA gene sequences showing the relationship between strain HUAS TT3^{T}, HUAS TT20^{T} and selected species of the genus *Streptomyces*. *Mycobacterium tuberculosis* H37Rv^{T} was used as an outgroup. Bootstrap percentages over 50% derived from 1000 replications are shown at the nodes. Scale bar, 0.0050 substitutions per site.

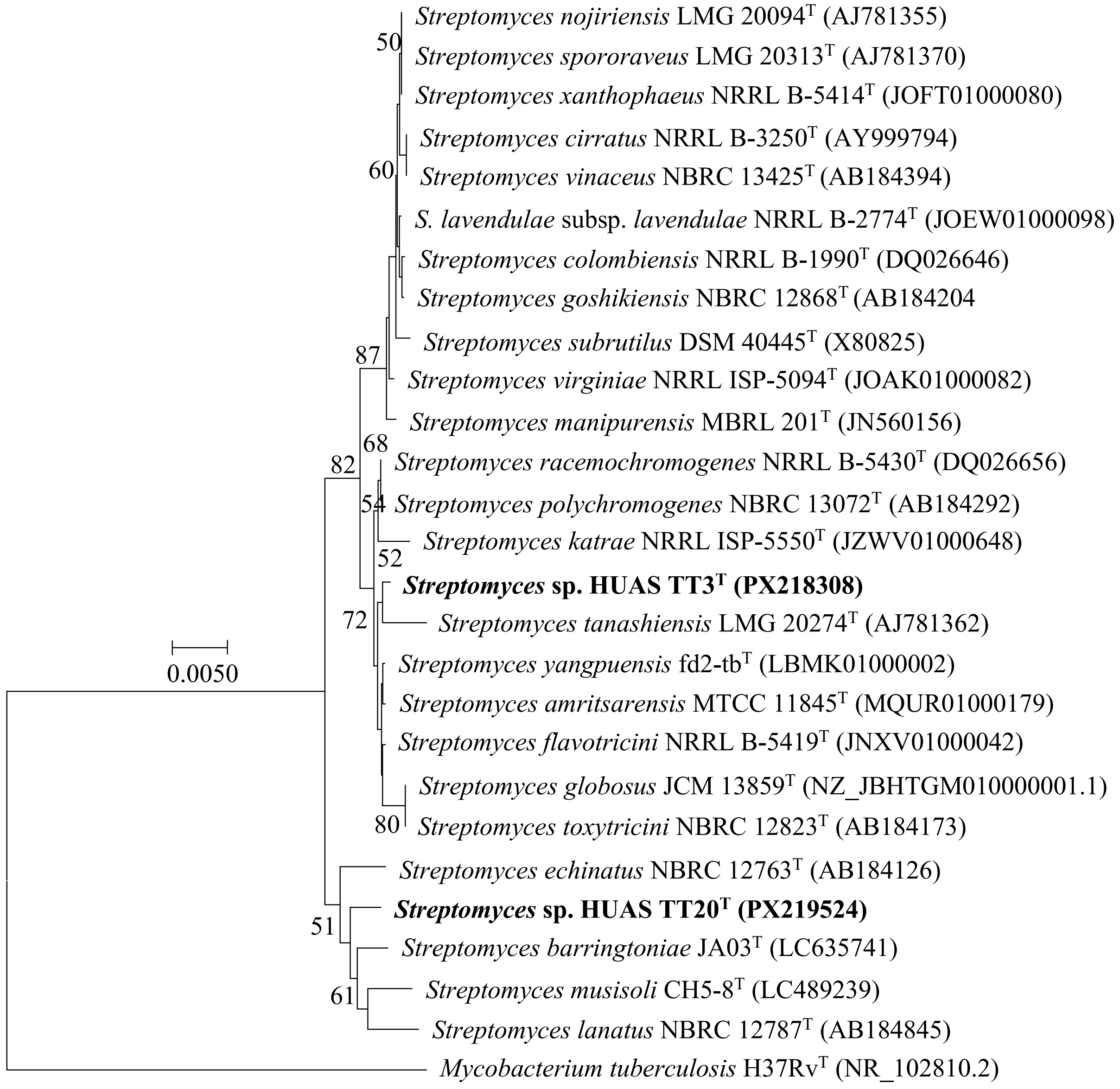

Fig. S4. Neighbour-joining phylogenetic tree based on five housekeeping gene sequences showing the relationship between strain HUAS TT3^T, HUAS TT20^T and selected species of the genus *Streptomyces*. *Mycobacterium tuberculosis* H37Rv^T was used as an outgroup. Bootstrap percentages over 50% derived from 1000 replications are shown at the nodes. Scale bar, 0.050 substitutions per site.

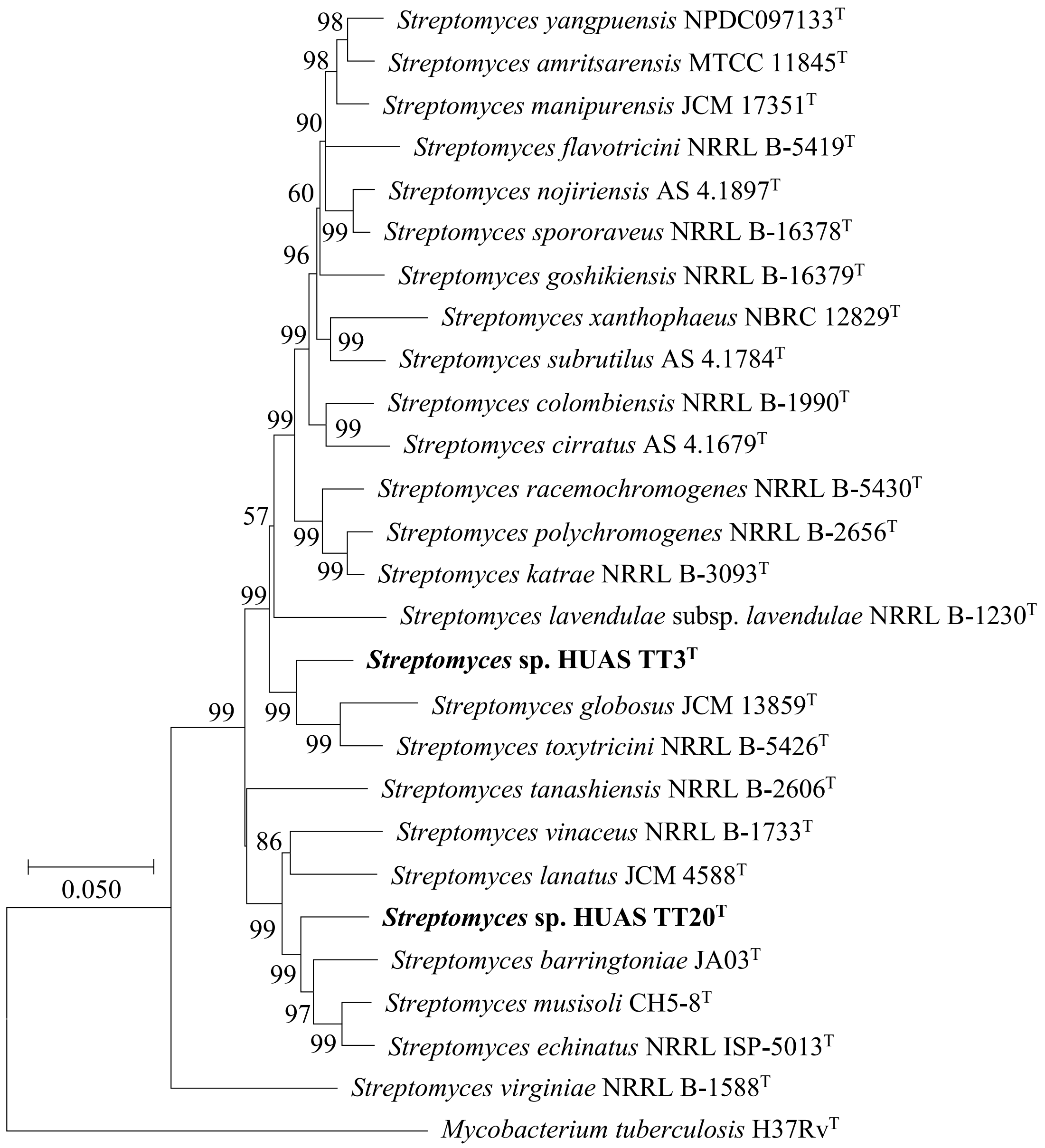

Fig. S5. Minimum evolution phylogenetic tree based on five housekeeping gene sequences showing the relationship between strain HUAS TT3^T, HUAS TT20^T and selected species of the genus *Streptomyces*. *Mycobacterium tuberculosis* H37Rv^T was used as an outgroup. Bootstrap percentages over 50% derived from 1000 replications are shown at the nodes. Scale bar, 0.050 substitutions per site.

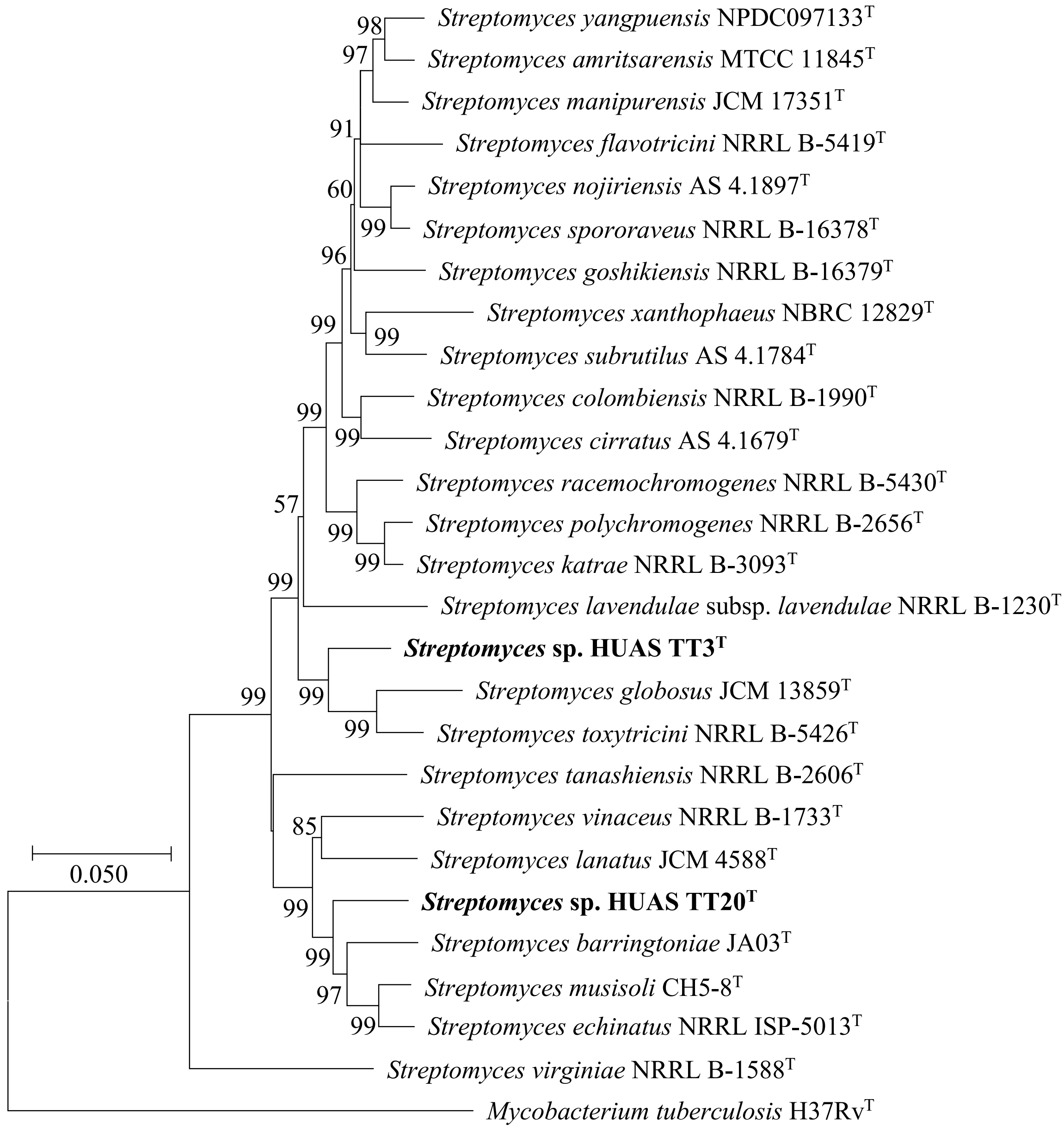

Fig. S6. Phylogenetic tree based on whole-genome sequences of strain HUAS TT3^{T}, strain HUAS TT20^{T} and their related reference strains. The tree inferred with FastME 2.1.6.1 (Farris, 1972, https://doi.org/10.1086/282802) from GBDP distances calculated from genome sequences. The branch lengths are scaled in terms of the GBDP distance formula d5. The numbers above the branches are GBDP pseudo-bootstrap support values of >60% from 100 replications, with an average branch support of 96.0%. The tree was rooted at the midpoint (Vincent et al., 2015, https://doi.org/10.1093/molbev/msv150).

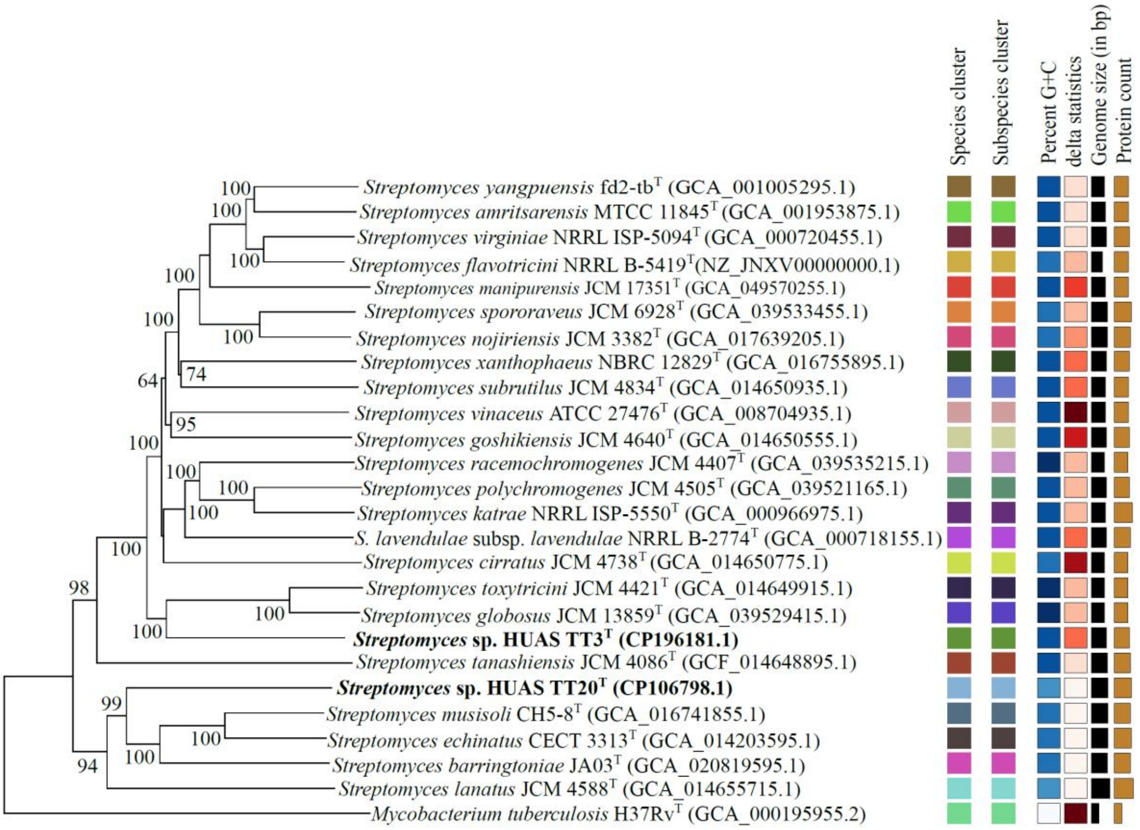

Table S1. Quality analysis and GenBank assembly of genomes of strain HUAS TT3^T, HUAS TT3^T and related reference strains.

No.	Species	GenBank assembly	COM (%)	CON (%)
1	*Streptomyces* sp. HUAS TT3^T	CP196181.1	99.43	2
2	*Streptomyces racemochromogenes* JCM 4407^T	GCA_039535215.1	99.57	0.57
3	*Streptomyces polychromogenes* JCM 4505^T	GCA_039521165.1	99.24	1.45
4	*Streptomyces yangpuensis* fd2-tb^T	GCA_001005295.1	99.62	1.29
5	*Streptomyces flavotricini* NRRL B-5419^T	NZ_JNXV00000000.1	97.06	0.71
6	*Streptomyces amritsarensis* MTCC 11845^T	GCA_001953875.1	99.81	0.38
7	*Streptomyces globosus* JCM 13859^T	GCA_039529415.1	99.66	0.47
8	*Streptomyces toxytricini* JCM 4421^T	GCA_014649915.1	99.91	0.09
9	*Streptomyces katrae* NRRL ISP-5550^T	GCA_000966975.1	92.85	1.7
10	*Streptomyces virginiae* NRRL ISP-5094^T	GCA_000720455.1	100	1.14
11	*Streptomyces tanashiensis* JCM 4086^T	GCA_014648895.1	99.91	1.33
12	*Streptomyces manipurensis* JCM 17351^T	GCA_049570255.1	99.91	1.33
13	*S. lavendulae* subsp. *lavendulae* NRRL B-2774^T	GCA_000718155.1	98.74	1.89
14	*Streptomyces xanthophaeus* NBRC 12829^T	GCA_016755895.1	99.87	2.02
15	*Streptomyces nojiriensis* JCM 3382^T	GCA_017639205.1	99.72	0.99
16	*Streptomyces sporoτaveus* JCM 6928^T	GCA_039533455.1	100	2.57
17	*Streptomyces cirratus* JCM 4738^T	GCA_014650775.1	99.81	2.18
18	*Streptomyces vinaceus* ATCC 27476^T	GCA_008704935.1	99.91	0.95
19	*Streptomyces goshikiensis* JCM 4640^T	GCA_014650555.1	99.91	1.49
20	*Streptomyces subrutilus* JCM 4834^T	GCA_014650935.1	99.53	0.44
21	*Streptomyces* sp. HUAS TT20^T	CP106798.1	97.82	0.76
22	*Streptomyces barringtoniae* JA03^T	GCA_020819595.1	99.62	0.8
23	*Streptomyces musisoli* CH5-8^T	GCA_016741855.1	100	1.54
24	*Streptomyces lanatus* JCM 4588^T	GCA_014655715.1	99.94	1.61
25	*Streptomyces echinatus* CECT 3313^T	GCA_014203595.1	99.86	0.82

Note: COM, completeness; CON, contamination.

Table S2. Cultural characteristics between strain HUAS TT3^T, *S. toxytricini* CGMCC 4.1734^T and *S. globosus* CGMCC 4.1969^T at 28 °C after incubation 21 days.

Characteristics	1	2	3
Color of aerial mycelium on No.1	White	White	Pink
Color of substrate mycelium on No.1	Shell Pink	White	Orange
Diffusible pigment on No.1	None	None	None
Color of aerial mycelium on R2A	Light color/White	White	Olive Green
Color of substrate mycelium on R2A	White	Citrine	Sudan Brown
Diffusible pigment on R2A	None	None	None
Color of aerial mycelium on ISP 2	Brownish Vinaceous	Salmon Buff	White
Color of substrate mycelium on ISP 2	White	Ochraceous Buff	Peach Red
Diffusible pigment on ISP 2	None	None	None
Color of aerial mycelium on ISP 3	Begonia Rose	Alizarine Pink	White
Color of substrate mycelium on ISP 3	Clay Color	White	White
Diffusible pigment on ISP 3	None	None	None
Color of aerial mycelium on ISP 4	White	Seashell Pink	Capucine Buff
Color of substrate mycelium on ISP 4	Citrine	White	Yellow Orange
Diffusible pigment on ISP 4	None	None	None
Color of aerial mycelium on ISP 5	White	White	Yellow Orange
Color of substrate mycelium on ISP 5	Pale Yellow Orange	White	Orange
Diffusible pigment on ISP 5	None	None	None
Color of aerial mycelium on ISP 6	Light Congo Pink	White	Victor Lake
Color of substrate mycelium on ISP 6	Buff-Pink	Pecan Brown	Carmine
Diffusible pigment on ISP 6	None	None	None
Color of aerial mycelium on ISP 7	Nude Pink	White	Mikado Orange
Color of substrate mycelium on ISP 7	White	White	Orange Pink
Diffusible pigment on ISP 7	None	None	None

Note: 1, HUAS TT3^T; 2, *S. toxytricini* CGMCC 4.1734^T; 3, *S. globosus* CGMCC 4.1969^T.No.1, Gause's synthetic No.1 medium; R2A, Reasoner'2A. All data were from this study.

Table S3. Cultural characteristics between strain HUAS TT20^T and *S. barringtoniae* LMG 32415^T at 28 °C after incubation 21 days.

characteristics	1	2
Color of aerial mycelium on No.1	Yellow	White
Color of substrate mycelium on No.1	Sulphur Yellow	Yellow
Diffusible pigment on No.1	None	None
Color of aerial mycelium on R2A	Gray	Grey
Color of substrate mycelium on R2A	Dresden Brown	Yellow
Diffusible pigment on R2A	None	None
Color of aerial mycelium on ISP 2	Cinnamon	Pink
Color of substrate mycelium on ISP 2	Light Buff	Yellow
Diffusible pigment on ISP 2	None	None
Color of aerial mycelium on ISP 3	Nude Pink	Yellow
Color of substrate mycelium on ISP 3	White	Yellow
Diffusible pigment on ISP 3	None	None
Color of aerial mycelium on ISP 4	White	White
Color of substrate mycelium on ISP 4	White	Grey
Diffusible pigment on ISP 4	None	None
Color of aerial mycelium on ISP 5	Primrose Yellow	White
Color of substrate mycelium on ISP 5	Light Cream Yellow	Orange Yellow
Diffusible pigment on ISP 5	Deep Olive Buff	None
Color of aerial mycelium on ISP 6	White	Light Greyish Brown
Color of substrate mycelium on ISP 6	Claret Brown	Light Greyish Brown
Diffusible pigment on ISP 6	Black	Brown
Color of aerial mycelium on ISP 7	Light Saimon Orange	Pale Pink
Color of substrate mycelium on ISP 7	Deep Olive	Deep Orange Yellow
Diffusible pigment on ISP 7	Black	None

Note: 1, HUAS TT20^T; 2, *S. barringtoniae* LMG 32415^T. No.1, Gause's synthetic No.1 medium; R2A, Reasoner'2A. All data were from this study.

Table S4. The fatty acid composition of strains HUAS TT3^T, *S. toxytricini* CGMCC 4.1734^T and *S. globosus* CGMCC 4.1969^T in trypticase soy broth (TSB) in shake flasks at 28 °C for 7 days.

Fatty acid (%)	HUAS TT3^T	CGMCC 4.1734^T	CGMCC 4.1969^T
$C_{10:0}$	3.2	0.7	1.6
$C_{10:0}$ 3-OH	0.8	0.5	0.6
$C_{11:0}$	1.2	Tr	Tr
anteiso-$C_{11:0}$	Tr	0.6	Tr
iso-$C_{12:0}$	ND	Tr	0.6
$C_{12:0}$ 3-OH	Tr	1.1	0.6
$C_{13:0}$	ND	1.3	1.0
$C_{13:0}$ 3-OH	0.5	Tr	Tr
$C_{14:0}$	4.2	6.5	4.4
anteiso-$C_{14:0}$	8.6	1.2	5.6
iso-$C_{14:0}$	2.3	Tr	1.2
iso-$C_{14:1}$ E	1.4	0.7	Tr
$C_{16:1}$ ω5c	15.6	5.6	7.6
anteiso-$C_{15:0}$	4.6	1.5	4.3
$C_{15:0}$	6.7	7.5	13.5
iso-$C_{15:0}$	4.1	3.3	3.6
iso-$C_{15:1}$ ω6c	ND	4.2	ND
iso-$C_{16:0}$	Tr	20.8	15.3
iso-$C_{16:0}$ 3-OH	0.6	Tr	Tr
iso-$C_{16:1}$ H	0.7	ND	Tr
iso-$C_{17:0}$ 3-OH	Tr	Tr	0.5
iso-$C_{16:0}$	9.3	16.3	16.7
$C_{16:0}$ 3-OH	ND	Tr	Tr
$C_{17:0}$	4.2	6.7	3.9
$C_{17:0}$ 2-OH	2.3	Tr	1
$C_{17:0}$ 10-methyl	1.6	Tr	0.6
Cyclo-$C_{17:0}$	2.3	4.3	3.3
anteiso-$C_{17:0}$	1.1	ND	ND
$C_{17:1}$ ω8c	10.2	8.4	5.7
Summed Feature 2	3.2	Tr	ND
Summed Feature 3	1.6	2.3	1.9
Summed Feature 9	1.7	Tr	ND

Note: All data were from this study. Tr, trace amount (<0.5%); ND, not detected. Summed Feature 2, aldehyde-$C_{12:0}$; Summed Feature 3, *iso* H-$C_{16:1}$/$C_{16:1}$ ω6c; Summed Feature 9, *iso*-$C_{17:1}$ ω9c/10-methyl $C_{16:0}$.

Table S5. The fatty acid composition of strain HUAS TT20^T and *S. barringtoniae* LMG 32415^T grown in TSB at 28 °C after incubation 7 days.

Fatty acid (%)	HUAS TT20^T	LMG 32415^T
iso-$C_{10:0}$	0.5	Tr
$C_{10:0}$	Tr	0.5
anteiso-$C_{11:0}$	ND	Tr
iso-$C_{12:0}$	0.7	Tr
$C_{12:0}$	Tr	0.6
iso-$C_{13:0}$	ND	Tr
anteiso-$C_{13:0}$	0.9	2.3
$C_{13:0}$	1.1	0.5
iso-$C_{14:0}$	17.1	10.2
$C_{14:0}$	1.6	9.7
$C_{13:0}$ *iso* 3OH	Tr	ND
anteiso-$C_{15:1}$ A	Tr	ND
iso-$C_{15:0}$	5.6	17.5
anteiso-$C_{15:0}$	8.7	12.5
iso-$C_{16:1}$ H	4.1	0.5
iso-$C_{16:0}$	22.9	10.6
$C_{16:0}$	7.1	19.7
anteiso-$C_{17:1}\omega9c$	0.4	0.5
iso-$C_{17:0}$	1.5	2.5
anteiso-$C_{17:0}$	3.7	4.3
$C_{18:0}$ 3OH	3.6	1.3
$C_{17:0}$	10.0	2.3
$C_{18:1}\omega9c$	Tr	Tr
$C_{18:0}$	0.2	0.5
Summed Feature 1	0.5	Tr
Summed Feature 3	5.4	Tr
Summed Feature 9	0.9	Tr

Note: Tr, trace amount (<0.5%); ND, not detected; Summed Feature 1, *iso*-$C_{15:1}$ H/13:0 3OH; Summed Feature 3, $C_{16:1}$ ω7c/$C_{16:1}$ ω6c; Summed Feature 9, *iso*-$C_{17:1}\omega9c$.

Table S6. Genome features of strains HUAS TT3^T, *S. toxytricini* JCM 4421^T and *S. globosus* JCM 13859^T.

Strains	1	2	3
Size (bp)	7,793,067	7,193,613	
Genes (total)	7,648	6,526	6,888
CDSs (total)	7,548	6,447	6,815
Genes (coding)	7,286	6,334	6,592
CDSs (with protein)	7,286	6,334	6,592
Genes (RNA)	100	79	73
rRNAs	24	5	5
complete rRNAs	24	2	4
tRNAs	73	71	65
ncRNAs	3	3	3
Pseudo Genes (total)	262	113	223
CDSs (without protein)	262	113	223
Pseudo Genes (ambiguous residues)	0	0	0
Pseudo Genes (frameshifted)	118	47	56
Pseudo Genes (incomplete)	199	89	180
Pseudo Genes (internal stop)	18	6	7
DNA G+C content	73.0%	73.8%	
Accession numbers	CP196181.1	GCA_000223905.1	GCA_039529415.1

Note: 1, HUAS TT3^T; 2, *S. toxytricini* JCM 4421^T; 3, *S. globosus* JCM 13859^T.

Table S7. Genome features of strains HUAS TT20^T and *S. barringtoniae* JA03^T.

Strains	1	2
Size (bp)	9,258,818	9,045,328
Genes (total)	8,788	8,243
CDSs (total)	8,693	8,164
Genes (coding)	8,145	7,827
CDSs (with protein)	8,145	7,827
Genes (RNA)	95	79
rRNAs	18	3
complete rRNAs	18	3
tRNAs	74	73
ncRNAs	3	3
Pseudo Genes (total)	548	337
CDSs (without protein)	548	337
Pseudo Genes (ambiguous residues)	0	0
Pseudo Genes (frameshifted)	273	127
Pseudo Genes (incomplete)	365	273
Pseudo Genes (internal stop)	32	18
DNA G+C content	70.5%	71.3%
Accession numbers	CP106798.1	GCA_020819595.1

Note: 1, HUAS TT20^T; 2, *S. barringtoniae* JA03^T.

Table S8. The subsystem category number of genes of strains HUAS TT3^T, *S. toxytricini* JCM 4421^T and *S. globosus* JCM 13859^T based on RAST annotation server.

Subsystem Feature Counts	1	2	3
Amino Acids and Derivatives	398	360	356
Carbohydrates	326	252	254
Protein Metabolism	247	223	218
Cofactors, Vitamins, Prosthetic Groups, Pigments	188	175	181
Nucleosides and Nucleotides	114	101	103
DNA Metabolism	90	93	100
Respiration	115	114	110
Membrane Transport	49	36	31
RNA Metabolism	55	46	52
Cell Wall and Capsule	48	40	40
Virulence, Disease and Defense	49	42	44
Fatty Acids, Lipids, and Isoprenoids	167	141	147
Stress Response	47	41	40
Sulfur Metabolism	10	6	6
Phosphorus Metabolism	29	23	23
Regulation and Cell signaling	30	20	23
Miscellaneous	30	27	30
Metabolism of Aromatic Compounds	19	15	14
Potassium metabolism	14	9	9
Iron acquisition and metabolism	28	30	31
Nitrogen Metabolism	17	19	22
Secondary Metabolism	6	12	9
Dormancy and Sporulation	1	1	1
Phages, Prophages, Transposable elements, Plasmids	1	1	2

Note: 1, HUAS TT3^T; 2, *S. toxytricini* JCM 4421^T; 3, *S. globosus* JCM 13859^T.

Table S9. The subsystem category number of genes of strains HUAS TT20^T and *S. barringtoniae* JA03^T based on RAST annotation server.

Subsystem Feature Counts	1	2
Amino Acids and Derivatives	452	434
Carbohydrates	407	418
Protein Metabolism	247	31
Cofactors, Vitamins, Prosthetic Groups, Pigments	213	211
Nucleosides and Nucleotides	107	125
DNA Metabolism	113	112
Respiration	146	142
Membrane Transport	49	44
RNA Metabolism	56	60
Cell Wall and Capsule	65	5
Virulence, Disease and Defense	60	54
Fatty Acids, Lipids, and Isoprenoids	224	191
Stress Response	71	72
Sulfur Metabolism	9	16
Phosphorus Metabolism	40	12
Regulation and Cell signaling	25	23
Miscellaneous	38	43
Metabolism of Aromatic Compounds	21	55
Potassium metabolism	15	242
Iron acquisition and metabolism	30	30
Nitrogen Metabolism	48	19
Secondary Metabolism	12	6
Dormancy and Sporulation	8	1
Phages, Prophages, Transposable elements, Plasmids	3	0

Note: 1, HUAS TT20^T; 2, *S. barringtoniae* JA03^T.

Table S10. The distribution of biosynthetic gene clusters in the genome of strain HUAS TT3^T, *S. toxytricini* JCM 4421^T and *S. globosus* JCM 13859^T by antiSMASH analyses.

	1	2	3
Terpene	8	6	7
T1PKS	7	0	4
NRPS	5	4	4
NRPS-like	4	2	4
NI-siderophore	3	3	3
Melanin	2	1	1
NAPAA	2	0	0
RiPP-like	2	0	5
T3PKS	2	2	2
Azole-containing-RiPP	1	1	2
Butyrolactone	1	2	2
Guanidinotides	1	1	1
hglE-KS	1	2	3
Hydrogen-cyanide	1	0	0
Indole	1	0	0
Lanthipeptide-class-i	1	1	0
Lassopeptide	1	0	0
Phosphonate	1	0	0
RRE-containing	1	0	1
T2PKS	1	1	1
Terpene-precursor	1	1	0
RiPP-like	0	5	1
Benzoxazole	1	0	1
CDPS	0	1	0
Hydrogen-cyanide	0	1	1
Lanthipeptide-class-ii	0	1	2
Lanthipeptide-class-iv	0	1	0
Linaridin	0	1	1
NRP-metallophore	0	1	0
Nucleoside	0	1	0
TransAT-PKS	0	0	1

Note: 1, HUAS TT3^T; 2, *S. toxytricini* JCM 4421^T; 3, *S. globosus* JCM 13859^T.T1PKS, Type I PKS (Polyketide synthase); NRPS, Non-ribosomal peptide synthetase; NRPS-like, NRPS-like fragment; NI-siderophore, NRPS-independent, IucA/IucC-like siderophores (siderophore prior to 7.0); NAPAA, Non-alpha poly-amino acids like e-

Polylysin; RiPP-like, Other unspecified ribosomally synthesised and post-translationally modified peptide product (RiPP); T3PKS, Type III PKS; Azole-containing-RiPP, (Thio)azol(in)e-containing peptides, linear and macrocyclic; Guanidinotides, Pheganomycin-style protein ligase-containing cluster; hglE-KS, Heterocyst glycolipid synthase-like PKS; Hydrogen-cyanide, Hydrogen cyanide (AF208523, doi:10.1128/jb.182.24.6940-6949.20); Lanthipeptide-class-i, Class I lanthipeptides like nisin; RRE-containing, RRE-element containing cluster; T2PKS, Type II PKS; Terpene-precursor, Compound likely used as a terpene precursor; Benzoxazole, Benzoxazoles; CDPS, tRNA-dependent cyclodipeptide synthases; Hydrogen-cyanide, Hydrogen cyanide (AF208523, doi:10.1128/jb.182.24.6940-6949.20); Lanthipeptide-class-ii, Class II lanthipeptides like mutacin II (U40620); Lanthipeptide-class-iv, Class IV lanthipeptides like venezuelin (HQ328852); Linaridin, Linear arid peptide such as cypemycin (HQ148718) and salinipeptin (MG788286); NRP-metallophore, Non-ribosomal peptide metallophores.

Table S11. The distribution of biosynthetic gene clusters in the genome of strain HUAS TT20^T and *S. barringtoniae* JA03^T by antiSMASH analyses.

	1	2
Terpene	8	9
T1PKS	5	12
NI-siderophore	4	2
NRPS	4	2
NAPAA	3	2
Redox-cofactor	3	0
RiPP-like	3	2
T3PKS	3	2
Butyrolactone	2	0
NRPS-like	2	0
Arylpolyene	1	0
Darobactin	1	0
Ectoine	1	1
hglE-KS	1	1
Lanthipeptide-class-ii	1	0
Melanin	1	2
Resorcinol	1	0
T2PKS	1	0
Terpene-precursor	1	3
TransAT-PKS	1	0
CDPS, hydrogen-cyanide	0	1
Lanthipeptide-class-iii	0	1
Lassopeptide, butyrolactone	0	1
Phenazine, thioamitides	0	1
Triceptides	0	1

Note: 1, HUAS TT20^T; 2, *S. barringtoniae* JA03^T. Redox-cofactor, Redox-cofactors such as PQQ (NC_021985:1458906-1494876); Darobactin, Darobactin-like compounds; Lanthipeptide-class-iii, Class III lanthipeptides like labyrinthopeptin (FN178622); Triceptide, Triceptides; CDPS, tRNA-dependent cyclodipeptide synthases; hydrogen-cyanide, Hydrogen cyanide (AF208523, doi:10.1128/jb.182.24.6940-6949.20); lassopeptide, Lasso peptide; butyrolactone, Butyrolactone; thioamitides, Thioamitide RiPPs as found in JOBF01000011.

Table S12. GenBank accession numbers of the sequences used in MLSA (multilocus sequence analysis).

No.	Strains	16S rRNA	*atp*D	*gyr*B	*rec*A	*rpo*B	*trp*B
1	HUAS TT3^T	PX218308.1	CP196181.1 2676204-2677652	CP196181.1 3983284-	CP196181.1 2283641-2284762	CP196181.1 3343494-3346976	CP196181.1 5643319-5644569
2	*S. racemochromogenes* NRRL B-5430^T	DQ026656	KT384698.1	KT385048.1	KT385399.1	KT389019.1	KT389367.1
3	*S. polychromogenes* NRRL B-2656^T	AB184292	KT384688.1	KT385038.1	KT385389.1	KT389019.1	KT389358.1
4	*S. yangpuensis* NPDC097133^T	LBMK01000002	NZ_JBIWDY010000001.1 505785-507227	NZ_JBFACO 37389-39410	NZ_JBFACO01000 33665-34780	NZ_JBFACO010000006.1 c281921-278439	NZ_JBFACO01000005 5753-7003
5	*S. flavotricini* NRRL B-5419^T	JNXV01000042	KT384554.1	KT384903.1	KT385252.1	KT388873.1	KT389223.1
6	*S. amritsarensis* MTCC 11845^T	MQUR01000179	NZ_MQUR01000001.1 c158717-157275	NZ_MQUR01 c10492-8471	NZ_MQUR010000 16469-17587	NZ_MQUR01000070.1 c7521-4039	NZ_MQUR01000005.1 30899-32149
7	*S. globosus* AS.4.320^T	AJ781330	NZ_BAAAMT010000019.1 c21210-19774	NZ_BAAAM c6594724-	NZ_BAAAMT010 1694208-1695332	NZ_BAAAMT010000019 2690237-2693719	NZ_BAAAMT010000 1853-3100
8	*S. toxytricini* NRRL B-5426^T	AB184173	KT384740.1	KY000587.1	KT385442.1	KT389060.1	KT389409.1
9	*S. katrae* NRRL B-3093^T	JZWV01000648	KT384608.1	KT384957.1	KT385307.1	KT388928.1	KT389277.1
10	*S. virginiae* NRRL B-1588^T	JOAK01000082	KT384515.1	KT384864.1	KT385212.1	KT388834.1	KT389184.1
11	*S. tanashiensis* NRRL B-2606^T	AJ781362	KT384656.1	KT385006.1	KT385357.1	KT388976.1	KT389325.1
12	*S. manipurensis* JCM 17351^T	JN560156	NZ_CP186950.1 5006363-5007805	NZ_CP18695 3720441-	NZ_CP186950.1 5415024-5416133	NZ_CP186950.1 4346776-4350258	NZ_CP186950.1 2023645-2024916
13	*S. lavendulae* subsp. *lavendulae* NRRL B-	JOEW01000098	KT384619.1	KT384968.1	KT385318.1	KT388939.1	KT389288.1
14	*S. xanthophaeus* NBRC 12829^T	JOFT01000080	NZ_BNEE01000006.1 c2796391-2794949	NZ_BNEE010 193038-	BNEE01000006.1 2386479-2387594	NZ_BNEE01000006.1 3467755-3471237	NZ_BNEE01000006.1 5897740-5898984
15	*S. nojiriensis* AS 4.1897^T	AJ781355	EF031320.1	EF055007.1	EF055062.1	EF055117.1	EF055172.1
16	*S. spororaveus* NRRL B-16378^T	AJ781370	KT384727.1	KT385076.1	KT385428.1	KT389047.1	KT389396.1
17	*S. colombiensis* NRRL B-1990^T	DQ026646	KT384772.1	KT385120.1	KT385474.1	KT389092.1	KT389441.1
18	*S. cirratus* AS 4.1679^T	AY999794	EF031301.1	EF054988.1	EF055043.1	EF055098.1	EF055153.1
19	*S. vinaceus* NRRL B-1733^T	AB184394	KT384468.1	KT384817.1	KT385165.1	KT388787.1	KT389137.1
20	*S. goshikiensis* NRRL B-16379^T	AB184204	KT384728.1	KT385077.1	KT385429.1	KT389048.1	KT389397.1
21	*S. subrutilus* AS 4.1784^T	X80825	EF031306.1	EF054993.1	EF055048.1	EF055103.1	EF055158.1

22	HUAS TT20^T	PX219524.1	CP106798.1 5980343-5981779	CP106798.1 4412256-	CP106798.1 6451839-6452963	CP106798.1 5138552-5142037	CP106798.1 c2312572-2311280
23	*S. barringtoniae* JA03^T	LC635741	NZ_JAJHNP010000018.1 127626-129062	NZ_JAJHNP0 c328887-	NZ_JAJHNP01000 c526110-524983	NZ_JAJHNP010000008.1 c322550-319065	NZ_JAJHNP01000001 c129518-128235
24	*S. musisoli* CH5-8^T	LC489239	NZ_JAERRH010000012.1 c77156-75720	NZ_JAERRH 245143-	NZ_JAERRH0100 c169677-168550	NZ_JAERRH010000004. 200156-203641	NZ_JAERRH0100000 222352-223635
25	*S. lanatus* JCM 4588^T	AB184845	NZ_BNBM01000003.1 532592-534028	NZ_BNBM01 c198095-	BNBM01000022.1 c148292-147156	NZ_BNBM01000015.1 c40996-37511	NZ_BNBM01000005.1 165565-166848
26	*S. echinatus* NRRL ISP-5013^T	AB184126	KT384539.1	KT384888.1	KT385236.1	KT388858.1	KT389208.1
27	*Mycobacterium tuberculosis* H37Rv^T	NR_102810	NC_000962.3 1465841-1467301	NC_000962.3 5240-7267	NC_000962.3 3049052-3051424 7	NC_000962.3 759807-763325	NC_000962.3 1811127-1812359

Table S13. MLSA distance values for selected strains in this study.

Strains	MLSA distance (Kimura two-parameter)													
	1	2	3	4	5	6	7	8	9	10	11	12	13	14
1														
2	0.072													
3	0.070	0.035												
4	0.074	0.058	0.066											
5	0.084	0.074	0.083	0.053										
6	0.070	0.057	0.065	0.025	0.043									
7	0.071	0.103	0.105	0.107	0.111	0.103								
8	0.056	0.082	0.082	0.094	0.092	0.085	0.048							
9	0.065	0.035	0.017	0.061	0.079	0.058	0.099	0.079						
10	0.146	0.145	0.144	0.144	0.150	0.137	0.170	0.164	0.141					
11	0.086	0.091	0.096	0.107	0.105	0.107	0.097	0.089	0.093	0.154				
12	0.067	0.052	0.056	0.029	0.054	0.030	0.098	0.084	0.050	0.134	0.101			
13	0.085	0.094	0.092	0.093	0.099	0.087	0.108	0.100	0.088	0.148	0.102	0.084		
14	0.104	0.084	0.092	0.076	0.080	0.074	0.101	0.117	0.090	0.149	0.123	0.070	0.097	
15	0.074	0.054	0.062	0.040	0.060	0.036	0.106	0.091	0.057	0.148	0.110	0.031	0.081	0.063
16	0.070	0.053	0.061	0.036	0.053	0.034	0.103	0.088	0.057	0.147	0.105	0.031	0.082	0.062
17	0.073	0.047	0.054	0.056	0.073	0.052	0.111	0.091	0.054	0.148	0.110	0.051	0.068	0.069
18	0.081	0.063	0.071	0.065	0.078	0.065	0.106	0.090	0.067	0.154	0.119	0.063	0.082	0.078
19	0.104	0.104	0.105	0.110	0.104	0.105	0.121	0.104	0.103	0.148	0.101	0.103	0.116	0.132
20	0.079	0.058	0.070	0.052	0.064	0.050	0.104	0.088	0.067	0.151	0.111	0.047	0.088	0.068
21	0.079	0.068	0.071	0.050	0.064	0.051	0.107	0.090	0.069	0.145	0.105	0.047	0.085	0.061
22	0.089	0.101	0.098	0.100	0.104	0.096	0.120	0.106	0.099	0.154	0.097	0.096	0.101	0.121
23	0.101	0.101	0.101	0.106	0.106	0.102	0.121	0.108	0.099	0.159	0.101	0.104	0.109	0.129
24	0.094	0.100	0.098	0.106	0.100	0.098	0.122	0.107	0.096	0.159	0.095	0.099	0.105	0.128
25	0.106	0.105	0.105	0.104	0.102	0.098	0.128	0.114	0.100	0.153	0.105	0.101	0.105	0.126
26	0.096	0.100	0.099	0.103	0.102	0.097	0.125	0.109	0.096	0.156	0.101	0.100	0.110	0.123
27	0.311	0.314	0.317	0.323	0.314	0.320	0.325	0.312	0.314	0.301	0.313	0.321	0.322	0.337

Table S13. (continued)

Strains	MLSA distance (Kimura two-parameter)											
	15	16	17	18	19	20	21	22	23	24	25	26
16	0.015											
17	0.044	0.044										
18	0.056	0.057	0.044									
19	0.114	0.113	0.114	0.112								
20	0.040	0.037	0.044	0.056	0.114							
21	0.044	0.046	0.057	0.055	0.117	0.049						
22	0.104	0.102	0.104	0.108	0.079	0.110	0.109					
23	0.110	0.109	0.112	0.108	0.074	0.110	0.110	0.059				
24	0.103	0.104	0.108	0.104	0.077	0.113	0.108	0.055	0.046			
25	0.103	0.102	0.107	0.103	0.072	0.110	0.108	0.070	0.071	0.071		
26	0.104	0.102	0.105	0.109	0.082	0.112	0.111	0.056	0.052	0.024	0.078	
27	0.324	0.321	0.322	0.328	0.297	0.324	0.328	0.311	0.305	0.308	0.322	0.306

Note: The strains 1-27 are the same as those of Table S12.
